# Genome-wide identification and Phylogenic analysis of kelch motif containing ACBP in *Brassica napus*

**DOI:** 10.1186/s12864-015-1735-6

**Published:** 2015-07-09

**Authors:** Nadia Haingotiana Raboanatahiry, Yongtai Yin, Li Chen, Maoteng Li

**Affiliations:** College of Life Science and Technology, Huazhong University of Science and Technology, Wuhan, 430074 China; Hubei Collaborative Innovation Center for the Characteristic Resources Exploitation of Dabie Mountains, Huanggang, 435599 China

**Keywords:** Acyl-coA binding proteins, kelch motif, *Brassica*, Evolution, Phylogeny, Chromosome map

## Abstract

**Background:**

Acyl-coA binding proteins (ACBPs) bind long chain acyl-CoA esters with very high affinity. Their possible involvement in fatty acid transportation from the plastid to the endoplasmic reticulum, prior to the formation of triacylglycerol has been suggested. Four classes of ACBPs were identified in *Arabidopsis thaliana*: the small ACBPs, the large ACBPs, the ankyrin repeats containing ACBPs and the kelch motif containing ACBPs. They differed in structure and in size, and showed multiple important functions. In the present study, *Brassica napus* ACBPs were identified and characterized.

**Results:**

Eight copies of kelch motif ACBPs were cloned, it showed that *B. napus* ACBPs shared high amino acid sequence identity with *A. thaliana, Brassica rapa* and *Brassica oleracea*. Furthermore, phylogeny based on domain structure and comparison map showed the relationship and the evolution of ACBPs within *Brassicaceae* family: ACBPs evolved into four separate classes with different structure. Chromosome locations comparison showed conserved syntenic blocks.

**Conclusions:**

ACBPs were highly conserved in *Brassicaceae*. They evolved from a common ancestor, but domain duplication and rearrangement might separate them into four distinct classes, with different structure and functions. Otherwise, *B. napus* inherited kelch motif ACBPs from ancestor conserving chromosomal location, emphasizing preserved synteny block region. This study provided a first insight for exploring ACBPs in *B. napus*, which supplies a valuable tool for crop improvement in agriculture.

**Electronic supplementary material:**

The online version of this article (doi:10.1186/s12864-015-1735-6) contains supplementary material, which is available to authorized users.

## Background

Acyl-coA binding proteins (ACBPs) bind long chain acyl-coA esters through an acyl-coA-binding domain, with very high affinity [1–3]. They are involved in the formation of long-chain fatty acyl-coA pools and act as an intracellular acyl-coA transporter [4–6]. ACBPs are possibly the transporters of synthesized fatty acids from plastid to endoplasmic reticulum, prior to the biosynthesis of triacylglycerol (TAG), principal compound of essential oils used for food, biofuel and many industrial applications. *Arabidopsis thaliana* ACBPs (*At*ACBPs) are divided in four classes, with six members that differ in structure, functions, expression, subcellular location and acyl-coA ester affinities [1, 2, 7–10]. Class I are the smallest ACBPs, usually called ACBP6, and contain 92 amino acids. ACBP6 are localized in the cytosol, where they have affinity to bind linoleyl-coA [10], they are expressed in all plants organs [11]. These small ACBPs are involved in intracellular binding and trafficking of phosphatidylcholine in plant phospholipid metabolism [11]. They maintain balance between acyl-coA and phosphatidylcholine to improve acyl exchange [12]. Class II correspond to the ankyrin repeats proteins (ACBP1 and ACBP2); they share the 76.9 % of identity and have respectively 338 and 355 amino acids. ACBP1 and ACBP2 are localized in the plasma membrane and in the endoplasmic reticulum [10], both of them are membrane proteins involved in membrane-associated acyl-coA transfer and metabolism functions [13], they bind linoleoyl-CoA and linolenoyl-CoA esters [14]. ACBP1 are highly expressed in seeds and siliques [7] and ACBP2 are expressed in roots, stems and flowers [15]. The class III ACBPs are commonly called “large ACBPs” or ACBP3, they contain 362 amino acids. ACBP3 have an extracellular location, they are highly expressed in siliques and young shoots [10]. ACBP3 bind arachidonyl-CoA with high affinity [2]. The kelch motif proteins are the class IV ACBPs (ACBP4 and ACBP5); these two copies share the 81.4 % of identity and have respectively 668 and 648 amino acids. ACBP4 and ACBP5 are located in the cytosol [10], where they have high affinity to bind oleoyl-coA. In presence of free oleoyl-coA that might inhibit the glucose-6-phosphate in the plastid, they can suppress synthesis dysfunction of starch and fatty acid [1]. They have more significant role in lipid transfer from the plastids to the endoplasmic reticulum [11, 16], and more roles for the biosynthesis of non-plastidial membrane lipids [17]. These kelch motif ACBPs are expressed in all tissues, with a higher expression of ACBP4 in roots and a lower expression in siliques. ACBP5 were found in young shoots and mature leaves but in a lower rate than ACBP4 [18].

*Brassica napus* is an important oil crop. It is a descendant lineage from hybridization between *Brassica rapa* and *Brassica oleracea. B. napus* 10-kDa ACBPs (*Bn*ACBPs) were identified twenty years ago [19]. Recent study revealed that they could change acyl-coA and TAG composition in seeds [20]. The kelch motif *At*ACBPs have many important functions. Besides their ability to maintain and transport oleoyl-coA from plastid to endoplasmic reticulum in plant metabolism, their structure that contains kelch domains, a protein-protein interaction site, makes them involved in plant stress responses [13, 18, 21, 22]. Due to the importance attributed to the kelch motif *At*ACBPs and those of small *Bn*ACBPs, it might be possible that the kelch motif *Bn*ACBPs share the same, or more, functions with them. Thus, the identification and characterization of *Bn*ACBPs could provide a basis for further studies about their functions. In this way, their exploitation can be useful in agriculture.

The present study aims to explore ACBPs in *B. napus.* This report contains four parts. Firstly, ACBPs were identified and characterized in *B. rapa, B. oleracea* and *B. napus*; amino acid sequence identities were compared. Secondly, *At*ACBPs and *Bn*ACBPs domain structures were analyzed. Thirdly, a phylogenetic analysis based on conserved domain was carried out. Finally, the comparison in chromosome locations of the kelch motif ACBPs was made.

## Results

### ACBPs in *B. rapa, B. oleracea* and *B. napus*

ACBPs were identified based on homology with *At*ACBPs. Similar to *At*ACBPs, they could be divided in four classes, with multiple copies (Table 1). Small ACBPs had 90 to 92 amino acids, ankyrin repeats ACBPs had 339 to 364 amino acids, large ACBPs had 361 to 381 amino acids and kelch motif ACBPs had 665 to 667 amino acids. Small ACBPs in *B. rapa* (*Br*ACBPs) and in *B. oleracea* (*Bo*ACBPs) had three copies each; however, small *Bn*ACBPs had only four copies. They were inherited from Bra033875 (BnaA05g36060D), Bra038439 (BnaA08g07670D), Bra023206 (BnaAnng25690D) and Bol038626 (BnaCnng15340D). Amino acid sequence comparison showed that small *Bn*ACBPs could share an average identity of 82.6 % with *At*ACBPs, 99.6 % with *Br*ACBPs, and 98.5 % with *B. oleracea* ACBPs *Bo*ACBPs (Table 2). Each of ankyrin repeats *Br*ACBPs and *Bo*ACBPs had two copies. Thus, four ankyrin repeats *Bn*ACBPs could share about 85.5 % of amino acid sequence identity with *At*ACBPs, 98.3 % with *Br*ACBPs, and 98.9 % with *Bo*ACBPs. In addition, two ankyrin repeats *Bn*ACBPs (BnaA02g10270D and BnaC02g44810D) might belong to ACBP1 subclass (~86.8 % of identity with *At*ACBP1), and two other ankyrin repeats *Bn*ACBPs (BnaA01g16660D and BnaC01g20440D) might be ACBP2 (~85.9 % of identity with *At*ACBP2). Four copies of large *Bn*ACBPs were inherited from four copies of large ACBPs of *B. rapa* and *B. oleracea*. They shared about 59.7 % of amino acid sequence identity with *At*ACBPs, 98.5 % with *Br*ACBPs, and 94.1 % with *Bo*ACBPs. Eight copies of kelch motif *Bn*ACBPs were cloned and sequenced. They are available on GenBank (accession: AIS76194 to AIS76201). Kelch motif *BnACBPs* were cloned from material grown in a semi-winter type area in China, they were the subject to further function analysis; but also, different lines SNP might exist as the material used for the sequencing of *B. napus* is not the same. These kelch motif *Bn*ACBPs were compared with six copies of putative kelch motif *Bn*ACBPs found in the database. Amino acid sequence identity was compared (Table 3). Both AIS76195 and AIS76200 shared 99.7 % of amino acid sequence identity with BnaA05g31780D, and 99.3 % of identity with Bra039439. However, AIS76195 shared 99.6 % of identity with AIS76200. Similarly, both AIS76196 and AIS76201 shared respectively 100 % and 97.6 % of identity with BnaAnng02420D, and 100 % and 97.6 % with Bol002733. They, however, shared 99.1 % of identity between them. Besides, AIS76194 and AIS76199 shared 97.1 % of identity and AIS76197 and AIS76198 shared 95.5 % of identity. These eight cloned copies of kelch motif *Bn*ACBPs could share amino acid sequence identity of about 88.25 % with *At*ACBPs, 98.17 % with *Br*ACBPs, and 98.4 % with *Bo*ACBPs. Otherwise, AIS76194, AIS76195, AIS76196, AIS76199, AIS76200 and AIS76201 could be ACBP4 (~90.53 % of identity with *At*ACBP4), and AIS76197 and AIS76198 could be ACBP5 (~81.4 % of identity with *At*ACBP5). These results indicated that ACBPs were conserved in *B. rapa, B. oleracea* and *B. napus*. Moreover, *Bn*ACBPs shared high amino acid sequences identity with *At*ACBPs, *Br*ACBPs and *Bo*ACBPs.

**Table 1:** ACBP in *A. thaliana, B. rapa, B. oleracea and B. napus.* An, Cn or Un correspond to unspecified chromosome location

Class	*A. thaliana*			*B. rapa*			*B. oleracea*			*B. napus*		
		Chr	Size (Aa)		Chr	Size (Aa)		Chr	Size (Aa)		Chr	Size (Aa)
Small	At1g31812	AT1	92	Bra033875	A5	92	Bol038626	C4	92	BnaAnng25690D	An	90
				Bra038439	A8	92	Bol027060	C8	92	BnaA05g36060D	A5	92
				Bra023206	A9	90	Bol005980	C5	90	BnaA08G07670D	A8	92
										BnaCnng15340D	Cn	92
Ankyrin	At4g27780	AT4	354	Bra026307	A1	364	Bol013113	C1	364	BnaA02g10270D	A2	342
repeats	At5g53470	AT5	338	Bra022656	A2	341	Bol017188	C6	339	BnaA01g16660D	A1	364
										BnaC02g44810D	C2	339
										BnaC01g20440D	C1	364
Large	At4g24230	AT4	366	Bra013778	A1	376	Bol009564	C1	370	BnaA01g13710D	A1	381
				Bra019240	A3	362	Bol042158	C6	361	BnaA03g46540D	A3	362
										BnaC01g16110D	C1	364
										BnaC07g38820D	C7	361
Kelch	At3g05420	AT3	668	Bra020582	A2	667	Bol012774	C2	667	AIS76194	A3	666
motif	At5g27630	AT5	648	Bra001147	A3	666	Bol001638	C5	667	(BnaA03g29000D)		
				Bra039439	A5	667	Bol002733	C1	665	AIS76195	A5	667
				Bra040219	An	665	Bol034106	C3	666	(BnaA05g31780D)		
										AIS76196	An	665
										(BnaAnng02420D)		
										AIS76197	C2	667
										(BnaC02g39790D)		
										AIS76198	A2	667
										(BnaA02g31230D)		
										AIS76199	C3	666
										(BnaC03g34240D)		
										AIS76200	A5	667
										(BnaA05g31780D)		
										AIS76201	Un	665

**Table 2:** Comparison of amino acid sequence identity in *At*ACBPs, *Br*ACBPs, *Bo*ACBPs and *Bn*ACBPs*.* Numbers are in percent (%)

Class	*Bn*ACBPs	*At*ACBPs		*Br*ACBPs				*Bo*ACBPs			
Small		At1g31812		Bra033875	Bra038439	Bra023206		Bol038626	Bol027060	Bol005980	
	BnaAnng25690D	**80.4**		83.9	87	**100**		84.9	84.8	97.8	
	BnaA05g36060D	**83.7**		**98.9**	94.6	84.9		97.8	92.4	84.9	
	BnaA08G07670D	**82.6**		93.5	**100**	87		92.4	97.8	87.4	
	BnaCnng15340D	**83.7**		96.7	92.4	84.9		**100**	90.2	84.9	
Ankyrin		At5g53470	At4g27780	Bra026307		Bra022656		Bol013113		Bol017188	
repeats	BnaA02g10270D	**86.1**	73.8	70.1		**98.8**		69.9		94.7	
	BnaC02g44810D	**87.5**	73.9	71.2		95		71		**100**	
	BnaA01g16660D	70.8	**84.3**	**97.8**		71.8		96.2		72.1	
	BnaC01g20440D	70.3	**84.3**	97.3		70.9		**97.8**		71.8	
Large		At4g24230		Bra013778		Bra019240		Bol009564		Bol042158	
	BnaA01g13710D	**62.9**		**97.4**		64.9		85.3		64.4	
	BnaA03g46540D	**58.5**		65.8		**99.7**		63.7		88.7	
	BnaC01g16110D	**58.6**		84.1		63		**94.9**		62.4	
	BnaC07g38820D	**59**		66.2		90.1		63.6		**93.4**	
Kelch		At3g05420	At5g27630	Bra020582	Bra001147	Bra039439	Bra040219	Bol012774	Bol001638	Bol002733	Bol034106
motif	AIS76194	**90**	75.7	75.6	**96.5**	90.6	89.7	76.3	90.4	90.4	95.9
	AIS76195	**91.8**	76.9	77.5	89.3	**99.3**	90.4	77.4	98.5	90.4	90.8
	AIS76196	**90.1**	75.5	75.3	89.5	90.3	98.2	75.3	90.4	**100**	90.3
	AIS76197	78.2	**81.6**	96.4	75.1	76.9	74.6	**99.6**	77.7	75.5	76.6
	AIS76198	77	**81.2**	**97.6**	73.5	76.8	74	95.4	77.1	74.3	75
	AIS76199	**88.8**	74.8	75	95.5	90	88.9	75.7	89.7	89.7	**96.4**
	AIS76200	**91.5**	76.5	77.4	89.2	**99.3**	90.1	77	98.2	90.6	90.7
	AIS76201	**91**	76	76.2	89.2	92.7	96.1	76.2	92.8	**97.6**	90.1

Numbers in bold are the highest percentage of identity.

**Table 3:** Comparison of amino acid sequence identity in kelch motif *Bn*ACBPs*.* Numbers are in percent (%)

	BnaA02g31230D	BnaA03g29000D	BnaA05g31780D	BnaAnng02420D	BnaC02g39790D	BnaC03g34240D
AIS76194	75	**96.7**	91	90.4	76	95.9
AIS76195	77.2	89.5	**99.7**	90.9	77.1	91.4
AIS76196	74.4	89.7	90.7	**100**	75.2	90.6
AIS76197	95.7	75.1	77.4	75.5	**98.4**	76.8
AIS76198	**99.9**	73.5	77.2	74.3	95.1	75.3
AIS76199	74.4	95.6	90.4	89.7	75.4	**96.7**
AIS76200	77.1	89.4	**99.7**	90.6	76.7	91.3
AIS76201	75.3	89.4	93.1	**97.6**	75.9	91

Numbers in bold are the highest percentage of identity.

### Domain structure of A*t*ACBPs and *Bn*ACBPs

**Fig. 1:**
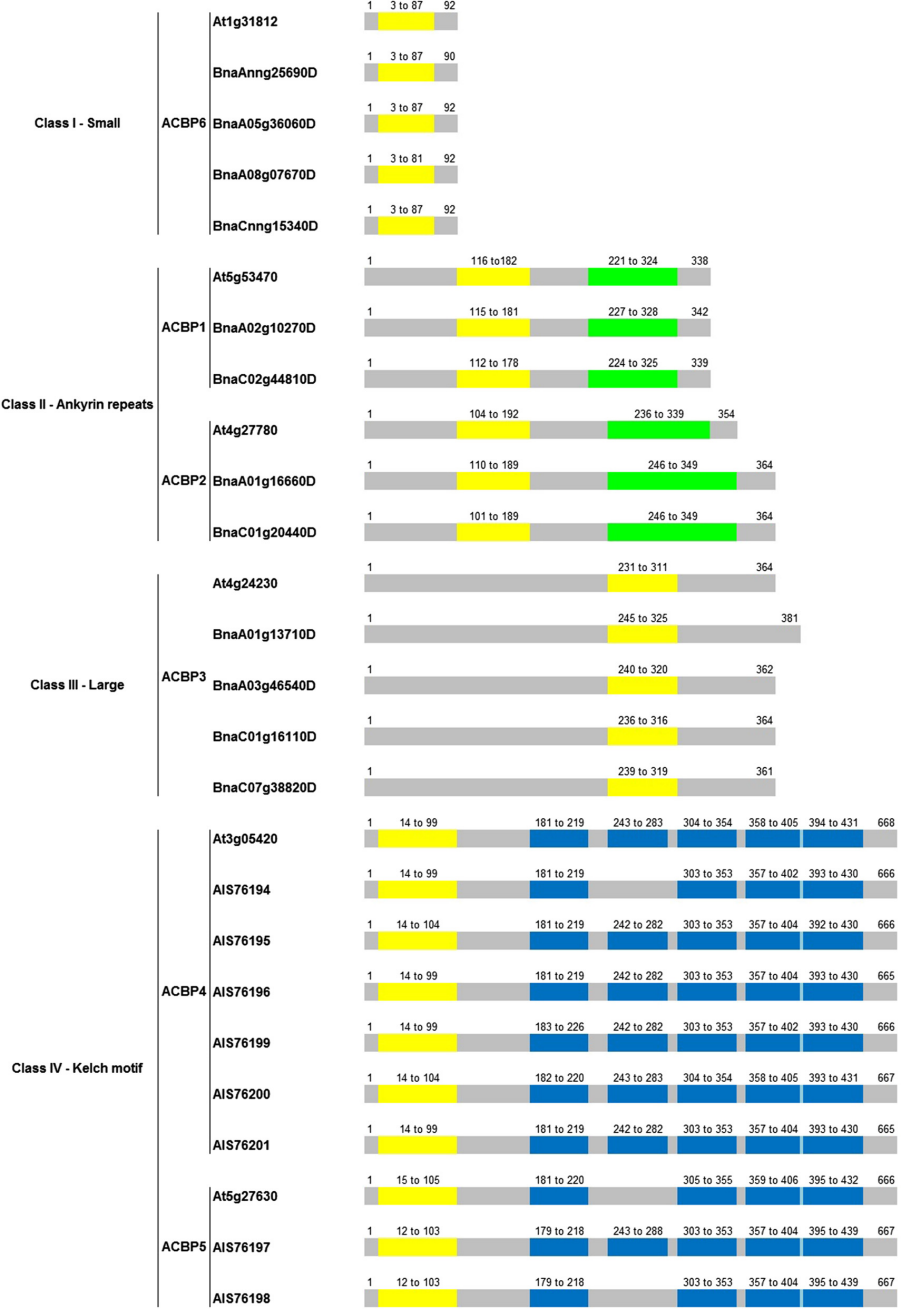
Domain structure of *At*ACBPs and *Bn*ACBPs. The architecture was generated by using Batch CD-search from NCBI database. ACBD are labeled in yellow, ankyrin repeats domains are in green and kelch domains are in blue

Domain structure of *At*ACBPs and *Bn*ACBPs were analyzed (Fig. 1). They commonly contained the acyl-coA binding domain (ACBD), but obviously differed in ACBD location and in extra domain structures in two classes of ACBP, the ankyrin repeats and the kelch motif ACBPs. ACBD was the only domain apparent in small ACBPs and seemed to be extended in almost all the protein (residue 3 to 87). Ankyrin repeats ACBPs had N-terminal ACBD (residue 112–182 in ACBP1 and 101–192 in ACBP2), and C-terminal ankyrin domain (residue 221–328 in ACBP1 and 236–349 in ACBP2). Similar to small ACBPs, large ACBPs had only one domain corresponding to ACBD (residue 231–325) located in the C-terminal of the proteins. However, ACBD were located in the N-terminal side of kelch motif ACBPs (residue 14–104 in ACBP4 and 12–105 in ACBP5). Additionally, four or five kelch motifs that belonged to Kelch 3 superfamily were found. Alignment of each kelch motif showed 40 highly conserved amino acid residues in the first kelch motif (181–226 in ACBP4, 179–220 in ACBP5), 29 residues in the second kelch motif (242–283 in ACBP4, 243–288 in ACBP5), 38 residues in the third kelch motif (303–354 in ACBP4, 303–355 in ACBP5), 38 residues in the fourth kelch motif (357–405 in ACBP4, 357–406 in ACBP5), and 36 residues in the fifth kelch motif (392–431 in ACBP4, 395–439 in ACBP5) (see Figure S1, Additional file 1). Apparently, the second kelch motif was missing in At5g27630, in AIS76194 and in AIS76198 (see Figure S1, Additional file 1-II). Alignment showed that At5g27630 differed from other ACBPs in Gln-256, Pro-272, Met-278, Cys-280 and Ser-282, likewise in AIS76194 and AIS76198, Phe-255 and Ile-280 were uncommon. Finally, the alignment of all four classes of ACBPs highlighted conserved amino acid residues, but only within the ACBD. Nevertheless, ACBD had some dissimilar amino acid sequences in each class of ACBP (see Figure S2, Additional file 2). These findings indicated ACBPs differed in domain structure, and *Bn*ACBPs and *At*ACBPs domain structure were very similar.

### Phylogenetic relationship of *Brassicaceae* ACBPs

**Fig. 2:**
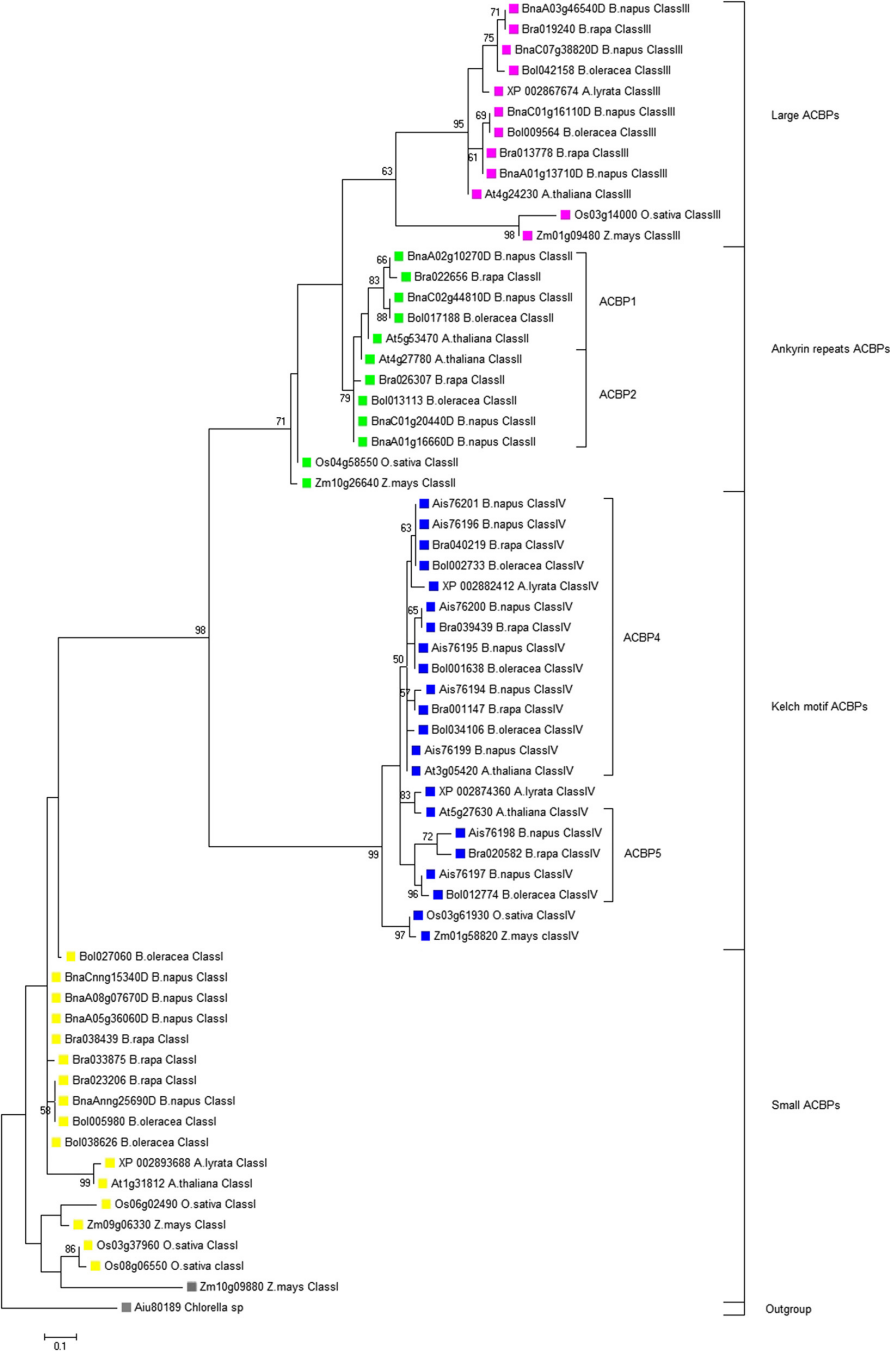
Evolutionary relationships of land plant ACBPs. The evolutionary history was inferred by using the Maximum Likelihood method based on Equal input model. The analysis involved 64 amino acid sequences. Evolutionary analyses were conducted in MEGA6

Analysis was carried out to highlight the phylogenetic relationships among *Brassicaceae* ACBP families. Five species belonging to *Brassicaceae* family and two Monocots species belonging to *Poaceae* family were used for the analysis. Each species could contain ACBPs belonging to the four classes, except for *A. lyrata* in which class II was missing. Sixty-four amino acid sequences were involved in the analysis. The tree was rooted with *Chlorella sp.* (Aiu80187) and was inferred based on domain structure using Neighbor-joining (not shown) and Maximum likelihood (Fig. 2), topologies were very similar. The tree was divided into four major clusters corresponding to separate classes of ACBP. The topology of the tree related to each class of ACBP was almost uniform: Monocots (*O. sativa* and *Z. mays*) diverged before the *Brassicaceae* family and appeared to be distantly related with them. Obviously, class I was basal, but lacked bootstrap support (BS < 50 %). Class I ACBPs with 90 amino acids (Bra023206, Bol005980 and BnaAnng25690D) diverged separately from those with 92 amino acids. Class IV appeared as a well supported clade (BS = 99 %), it originated after the divergence of Class I. ACBP5 diverged before ACBP4; similarly in Class II, ACBP2 diverged before ACBP1. Class II was moderately supported (BS = 71 %). Class III, however, was weakly supported (BS = 63 %). As in Class I, Class III shorter proteins (Bra019240, Bol042158, BnaA03g46540D and BnaC07g38820D) diverged separately from the longer ones. These results illustrated the evolutionary relationship of ACBPs, and their divergence leading to four distinct classes.

### Chromosome location of kelch motif *Bn*ACBPs

**Fig. 3:**
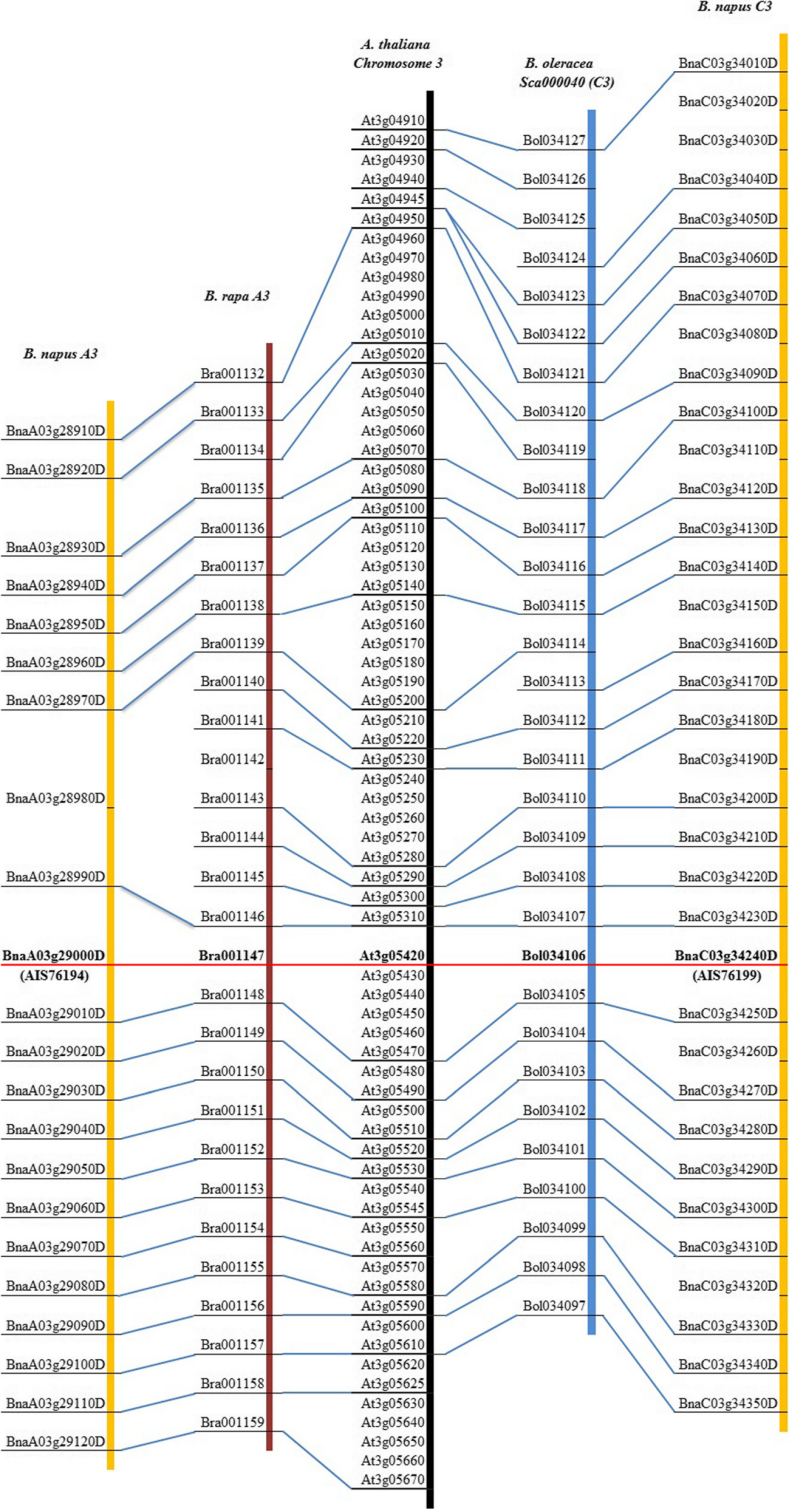
Comparison maps of kelch motif *At*ACBP, *Br*ACBP, *Bo*ACBP and *Bn*ACBP. Maps were built manually, associating the homologue genes from each species. ACBPs are in bold. *B. napus* chromosomes are in yellow: this figure represents AIS76194 and AIS76199

In our previous analysis, eight copies of kelch motif *Bn*ACBPs were cloned and they corresponded to six copies of kelch motif *Bn*ACBPs found on the database. These six copies of kelch motif *Bn*ACBPs were located on six different chromosomes and they were associated to ancestors *Br*ACBPs, *Bo*ACBPs and *At*ACBPs. Four maps were constructed. BnaA03g29000D (AIS76194) was located on A3, similar to parent Bra001147 (Fig. 3). BnaC03g34240D (AIS76199) was located on C3; parent Bol034106 was placed on Sca000040 (Fig. 3). To investigate where each chromosome was located on the scaffold, we used the blastp search of EnsemblPlants by EBI (http://plants.ensembl.org/) [23], with Bol034106 as query. Thus, Bol034106 is on Sca000040 of C3. As well, BnaAnng02420D (AIS76196) had homolog Bol002733 placed on Sca000378 of C1 (see Figure S3, Additional file 3). BnAnng02420D should have been found on C1, but obviously was located on an unspecified chromosome A. BnaA05g31780D (AIS76195/AIS76200) and parent Bra039439 were both placed on A5 (see Figure S4, Additional file 4). BnaC02g39790D (AIS76197) and homolog Bol012774 were both located on C2, whereas BnaA02g31230D (AIS76198) and Bra020582 were both placed on A2 (see Figure S5, Additional file 5). Chromosome location of *Bn*ACBP-AIS76201 could not be predicted, considering amino acid identity it could share with available genes on the database, it might be on a missing genomic contig. In this study, genes could be related in ascendant or descendant order but synteny was still preserved. Otherwise, one gene in *A. thaliana* might have two copies homolog on the same chromosome in *Brassica*, also one gene copy in *B. rapa o*r *B. oleracea* might have two copies homolog in *B. napus*. For example, At3g05545 had two copies homolog to Bra039429 and Bra039430 on A5 (see Figure S4, Additional file 4). However, one copy of *Br*ACBP or *Bo*ACBP had only one copy of *Bn*ACBP homolog on the related chromosome location. As predicted ACBP4, BnaA03g29000D (AIS76194), BnaC03g34240D (AIS76199), BnaA05g31780D (AIS76195/AIS76200) and BnaAnng02420D (AIS76196) were associated to homolog At3g05420 (*At*ACBP4) located on chromosome 3. As well, predicted ACBP5 BnaC02g39790D (AIS76197) and BnaA02g31230D (AIS76198) were associated to At5g25630 (*At*ACBP5). These findings suggested *B. napus* inherited ACBP from *B. rapa* and *B. oleracea*, conserving their chromosome location.

## Discussion

### ACBPs were conserved in *Brassicaceae*

*Br*ACBPs, *Bo*ACBPs and *Bn*ACBPs were identified based on homology to *At*ACBPs. Their sizes were ranged from 90 to 667 amino acids, which were relatively close to those of *At*ACBPs with 92 to 668 amino acids [16]. Besides, eight copies of kelch motif ACBP were cloned from *B. napus* of a semi winter-type growing area in China. However, only six copies were found on the database given that two cloned copies (AIS76195 and AIS76200) shared the same amino acid sequence identity of 99.7 % with one copy of the database (BnaA05g31780D), and one cloned copy (AIS76201) could only share its highest identity (97.6 %) with BnaAnng02420D, which already shared 100 % of identity with AIS76196. This might be explained by the difference in plant materials. The *Brassica napus* French homozygous winter line ‘Darmor-bzh’ was used as reference for the genome sequencing [24]. The number of ACBP copies in *B. napus* obviously increased because *B. napus* inherited ACBP copies from its parents *B. rapa* and *B. oleracea*. Therefore, *Bn*ACBPs could be divided into four classes. *Bn*ACBPs shared an amino acid identity of 58.5 % to 91.8 % with *At*ACBPs, with the lowest rating in large ACBPs and the highest in the kelch motif ACBPs. Four small *Bn*ACBPs were acquired from the Genoscope Database. Earlier, six genes of about 90 amino acids in *Bn*ACBPs were identified, in which three genes were inherited from *B. rapa* and three genes were from *B. oleracea* [19]. The missing two copies corresponded to those inherited from *B. oleracea* (Bol027060 and Bol005980) given that they were not clearly specified from the database. Thus, small *Bn*ACBPs shared the 80.4 % to 83.7 % of amino acid sequence identity with *At*ACBPs, closely similar to previous finding in which the amino acid identity between small *Bn*ACBP and *At*ACBP was 84 % [9, 19]. *Bn*ACBPs shared high amino acid identity with *At*ACBPs, except for large ACBPs in which similarity was rather low compared to those of the other classes. Nevertheless, comparing large *Bn*ACBPs with homologues *Br*ACBPs and *Bo*ACBPs exposed high amino acid sequence identity 93.4 % to 99.7 %. Substantial mutations might have occurred during evolution resulting in a significant difference in amino acid sequence identity between these large *At*ACBPs and *Bn*ACBPs. Two classes of *At*ACBPs had two members: ankyrin repeats ACBPs and kelch motif ACBPs. Ankyrin repeats ACBPs could be subdivided in ACBP1 and ACBP2, they shared the 76.9 % of identity [1]. Similarly, kelch motif *At*ACBPs included ACBP4 and ACBP5, which shared the 81.4 % of identity [1]. Comparison in amino acid sequence identity allowed to define these subdivisions within the ankyrin repeats *Bn*ACBPs and the kelch motif *Bn*ACBPs. These findings affirm the conservation of ACBPs in *Brassicaceae,* with high amino acid sequence identity.

### *Bn*ACBPs differed in domain structure, similar to *At*ACBPs

Conserved domain in *At*ACBPs and *Bn*ACBPs were analyzed through batch CD-search of NCBI. ACBPs common domain structure was the acyl-coA binding domain (ACBD), which allowed them to fulfill their roles in binding acyl-coA esters with very high affinity [1–3]. *Bn*ACBPs structure conserved this ACBD but locations were different, as in *At*ACBPs. Moreover, in *A. thaliana* each class of ACBP had dissimilar affinity in binding acyl-coA as introduced previously. This dissimilarity might probably due to difference in amino acid sequence (see Figure S2, Additional file 2). Ankyrin repeats and kelch motif ACBPs differed from small and large ACBPs by the presence of protein-protein interaction site as additional domains [13, 14, 18, 21, 25]. One ankyrin domain was found in each protein. This domain of about 33 amino acid residues was involved in cell signals or regulation [25, 26]. Four or five kelch motifs were found in our analysis; previous findings reported five kelch domains in *At*ACBPs [1, 10]. Kelch motif is a sequence of 44 to 55 amino acids in the protein; it is one of ancient and ordinary domains, and usually a group of four to seven motifs forms the kelch repeat domain [27, 28]. In our analysis, loss of one kelch motif (residue 242 to 283) was observed in At5g25630, AIS76194 and AIS76198. This loss might be explained with amino acid substitutions in this region of protein (see Figure S1, Additional file 1). Function or expression of protein might be different within ACBPs, in consequence of this loss. For instance, *At*ACBP4, which contained five kelch motifs were highly expressed in root whereas A*t*ACBP5, which was demonstrated in our study to have only four kelch motifs, was expressed in shoots and mature, leaves [18]. These five kelch domains belonged to the Kelch 3 superfamily, but conserved amino acid residues were not identical, confirming the existence of multiple families of kelch domain [27, 28]. Ankyrin repeats and kelch motif domains could imply these ACBPs in many biological functions such as plant defense and stress responses [18, 22]: ankyrin repeats *At*ACBPs were involved in response to heavy metal accumulation [14] but also in tolerance of dry conditions [29]. Kelch motif *At*ACBP4 could act in response to accumulation of Pb (II) in root [30]. These findings confirm *Bn*ACBPs differ not only in size but also in domain structure.

### ACBPs evolved into four separate classes of diverse structure

ACBPs from a selected group of *Brassicaceae* and Monocots were assembled for the phylogenetic analysis. To emphasize relationship among them, both NJ and ML tree were reconstructed, based on the conserved domain. Sixty-four ACBPs were involved, including ACBP of algae to root the tree. The presence of ACBPs in these species indicated their importance in biological function, and their existence before the divergence of land plants [31, 32]. One class might have more than one copy due to duplication event resulting in dissimilar sequences [33]. In Monocots, class I contained two or three copies and the other classes contained only one copy, this shows that gene duplication occurred only in class I for this group [32]. *A. lyrata* Class II was missing. *A.lyrata* and *A. thaliana* are close enough that many traits are shared between them. Their divergence occurred about ten million years ago. *A. lyrata* genome is twice larger than that of *A. thaliana*. The missing Class II ACBPs in *A. lyrata* might have resulted from the insertions and deletion events that occurred in this evolution [34], leading to the loss of ankyrin repeats in *A. lyrata* ACBP. Monocots were distantly related to the *Brassicaceae* family, their divergence occurred earlier, during the evolution of land plants. In all ACBP classes, *Brassica* genera and *Arabidopsis* genera phylogenetic positions were close affirming that *Brassica* ACBPs shared common ancestry with *Arabidopsis* [35, 36]. The tree illustrated four major clusters corresponding to separate ACBP classes. Class I might be basal, since phylogenetic analysis with rice ACBPs suggested Class I might be ancestral [32], which is also accurate in our study. However, class I had inappropriate bootstrap support, which makes this hypothesis uncertain. Class I are single-domain proteins of about 90 amino acids, they were first cloned from *B. napus* [19]. Class I was demonstrated to be highly conserved in all species, not only in plants [37]. Class II and Class IV deviated separately after Class I divergence. In our analysis, ACBP1 and ACBP2 were distantly related, suggesting their early divergence, similarly to ACBP4 and ACBP5 of Class IV. Both Class II and Class IV are multi-domain proteins, which might be born from a pre-existing protein domain combination, since domains tend to combine within each other to generate new multi-domain proteins [38–40]. Class III originated after class II divergence; they had an N-terminal transmembrane domain as class II. Due to their phylogenetic position, class II and class III ACBPs were suggested to be functionally related [32]. ACBP is highly conserved in plants and paralogues born from a common ancestor [37]. ACBPs become larger with evolution [32]. This might explain the difference in size of ACBPs, smaller in the proposed ancestral Class I and larger in the other classes. In fact, protein domains as conserved in ACBPs are basic building blocks that define structure and function of proteins [40]; they have an independent evolution [41–43]. They originated from conglomerates of short polypeptide segments that fold together [44]. Duplication, divergence and recombination of domains generate proteins. New genes are mainly harbored from duplication and this could happen also for domains [42, 45]. Thus, evolution of ACBP by domain duplication and recombination might be possible. Duplication induced mutations explaining divergence; and recombination harbored new folds of new proteins with new functions that evolved from common ancestors [40, 46]. Consequently, protein domains change within sequence, structure and function but also in occurrence in the genomes of different organisms [43, 47]. Because duplication, convergence or divergence of protein domains [48–50], or formation of multi-domain proteins (as Class II and Class IV ACBPs) through domain combination [51, 52] are usual in protein evolution, this might have happened in evolutionary history of ACBP. Otherwise, ACBPs were demonstrated to show high evolutionary conservation in structure and function, precisely in basal cellular functions [31]. ACBPs were detected in different species: 30 % of residues among them are conserved in the acyl-coA binding domain (ACBD) [53]. Alignment of the four classes of ACBPs in our study confirmed ACBD as the only common domain; remaining amino acid residues had no similarities. Nevertheless, ACBDs showed some dissimilarity, which could explain diverse acyl-coA-binding preferences of different classes of ACBP [33, 43]. These findings confirm the high conservation of ACBPs in land plants and their diversity as consequences of evolution.

### *B. napus* inherited the kelch motif ACBPs from *B. rapa* and *B. oleracea*

Previously, our results demonstrated the conservation of ACBP in *Brassicaceae.* The kelch motif *Bn*ACBPs were demonstrated to conserve the same chromosome location as parents *Br*ACBPs and *Bo*ACBPs, which homologues in *A. thaliana* were located on chromosomes 3 or 5. In *A. thaliana,* chromosomes are subdivided into 24 blocks [54]. *At*ACBPs are located on block F of chromosome 3 and on block Q of chromosome 5. These blocks F and Q have corresponding blocks identified in *B. napus* [55] affirming their conservation in *Brassica* genera. Otherwise, gene loss and gene deletion possibly occurred within *B. napus* genome [24]. In our study, genes might appear to not have homolog in progenitors. This is the case of BnaA03g28980D and BnaC03g34150D which homolog was absent in A3 of *B. rapa* and C3 of *B. oleracea,* respectively (Fig. 3). Gene deletion might have occurred. Inversely, *B. rapa* and *B. oleracea* might not have homologues in *B. napus*. This is the case of Bra001142 and Bol034126 (Fig. 3), where gene loss might have occurred. Besides, genes could be present in *A. thaliana* but lacking in *Brassica* genera or inversely absent in *A. thaliana* but present in *Brassica* genera (case of Bol034113 on Fig. 3), as it was demonstrated that *Brassica* genome showed mixed losses and insertions compared with *A. thaliana* [55, 56]. Lastly, BnaAnng02420D (AIS76196), which is the homologue of Bol002733, was not found on chromosome C in *B. napus*, but on an unspecified chromosome A (see Figure S3, Additional file 3). BnaAnng02420D might be a converted gene, as many converted genes were found in *B. napus* genome [24]. However, the analyzing of neighbor genes showed that Bol002724 had homolog BnaCnng24690D on an unspecified chromosome C, Bol002722 had homolog BnaC03g46060D on C3 and Bol002738 had homolog BnaC05g33680D on C5, suggesting a misassembly of this region. These results confirm that synteny is preserved and kelch motif ACBPs are faithfully transferred to *B. napus*.

### Overview on the evolutionary history of *Brassica* genomes, based on ACBPs

The allotetraploid *B. napus* (AC, n = 19) was synthesized from hybridization between diploid parents *B. rapa* (A, n = 10) and *B. oleracea* (C, n = 9). The current study focused on ACBPs in *B. napus*, but their identification was made with homology based on *A. thaliana, B. rapa* and *B. oleracea* ACBPs, which could highlight the evolutionary history of *Brassica* genomes. ACBPs were conserved in *Brassicaceae.* The descendant line *B. napus* inherited 4 copies each of class I, class II and class III ACBPs, and 6 copies of kelch motif ACBPs (exclusive of results obtained from cloning). In total, 18 copies of ACBPs were inherited from parents *B. rapa* and *B. oleracea* and presented high amino acid sequence identity, similar structure and chromosome location with them. As 11 copies of ACBPs were found in each of *B. rapa* and *B. oleracea* (22 copies in total), four copies were lost in *B. napus*. They corresponded to two class I *Bo*ACBPs (Bol027060 and Bol005980), one class IV *Bo*ACBP (Bol001638) and one class IV *Br*ACBP (Bra040219) (see Figure S6, Additional file 6). Besides, kelch motif ACBPs were faithfully transferred to *B. napus*, with six different chromosomal locations similar to parents *B. rapa* and *B. oleracea*. Genomic block F and Q, which contain kelch motif *At*ACBPs were also in *B. napus*. The chromosome evolution of *Brassica* plants was studied through these genomic blocks, as reviewed by Cheng et al. [57]. They emphasized that whole genome triplication (WGT) promoted the diversification of *Brassica* plants. Genome comparison between *A. thaliana* and *B. rapa* or *B. oleracea* exposed this WGT event experienced by these two Brassica plants [58, 59]. In fact, *Brassicaceae* genomes are composed of 24 genomic blocks, the basic units of ancestral chromosome that reshuffled to generate the present day species. Then, three sets of 24 genomic blocks should exist in *B. rapa* and *B. oleracea*, which means 3 blocks F and 3 blocks Q each. This explains the increasing copies of kelch motifs ACBPs in *B. rapa* and *B. oleracea* (4 copies each). 3 blocks F and 1 block Q, in each of *B. rapa* and *B. oleracea,* contain these kelch motif ACBP. However, 6 copies were inherited by *B. napus*, 2 copies were lost. These 6 conserved copies were on 4 blocks F (A3, A5, C1 and C3) and 2 blocks Q (A2 and C2). *B. napus* conserved these 6 genomic blocks from *B. rapa* and *B. oleracea*. These finding illustrate the evolutionary history of *Brassica* genomes, using ACBPs as reference.

## Conclusion

*Brassica napus* ACBPs were identified and compared to ACBPs of relatives *A. thaliana, B. rapa* and *B. oleracea*. Their close similarity is not surprising, no major abnormality was found. ACBPs had multiple copies. As domain proteins, they originated from the same ancestor that experienced domain duplication and rearrangement to generate new proteins with different stucture. This study is a prelude for ACBP investigation in *B. napus. *Multiple copies were found, multiple interesting functions are expected for crop improvement purpose.

## Methods

### Identification of *Br*ACBPs and *Bo*ACBPs

Identification of *BrACBPs and BoACBPs* was based on homology to *A. thaliana* ACBP proteins. *A. thaliana* ACBP (*At*ACBPs) were acquired from the *Arabidopsis* Information Resource - TAIR (http://www.arabidopsis.org) TAIR [60]. They were used as query to search for *Br*ACBPs and *Bo*ACBPs in the *Brassica* database - BRAD (http://brassicadb.org) [61].

### Research of *Bn*ACBPs

Kelch motif ACBPs were cloned from *B. napu*s that were grown for 60 days in Huazhong University of Science and Technology, Wuhan, China (semi winter-type rapeseed growing area). The oil content of the material was about 50 %. We conducted RNA extraction from siliques, by using Trizol RNA isoplus (Takara). To synthesize the first cDNA strand, RevertAid First Strand cDNA Synthesis Kit by Thermo Scientific was used. Primers were designed based on kelch motif *Br*ACBPs and *Bo*ACBPs sequences. Oligo7 software was operated to design these primers (sequences in Table S1, Additional file 7). The amplification was performed with KOD enzyme and then with ES Taq enzyme. The targeted genes were purified by CWBIO Gel extraction kit. They were then inserted into T-vector PMD19 by Takara Bio Inc. and integrated into *E. coli* DH5- α. Genes from positive colonies were consequently sequenced. Vector NTI Advanced 11 software (Invitrogen Corporation) was used to align the obtained sequences with CDS of *Br*ACBPs and *Bo*ACBPs to acquire CDS of *Bn*ACBPs, which were translated into protein sequences. Additionally, all four classes of *Bn*ACBPs were acquired from the CNS-Genoscope database (http://www.genoscope.cns.fr/brassicanapus/) [24] with *Br*ACBPs and *Bo*ACBPs as query.

### Comparison in amino acid sequence identity

Two comparisons were made. The first one was between cloned kelch motif *Bn*ACBPs compared with those acquired from database, and the second one was between *At*ACBPs and *Bn*ACBPs. Amino acid sequence identity was calculated by using EMBOSS Needle (http://www.ebi.ac.uk/Tools/psa/emboss_needle/) [62].

### Conserved domain analysis

Conserved domain in *At*ACBPs and *Bn*ACBPs were analyzed by using Batch CD-search tool in NCBI database, with E-value cut off 0.10 (http://www.ncbi.nlm.nih.gov/Structure/bwrpsb/bwrpsb.cgi) [63].

### Phylogenic analysis based on domain

ACBPs of selected species belonging to *Brassicaceae* family were used for the phylogenic analysis: *Arabidopsis thaliana* (Thale cress), *Arabidopsis lyrata* (Lyre-leaf rockcress), *B. rapa* (Field mustard), *B. oleracea* (Broccoli) and *B. napus* (Rape). Two Monocots were added to the tree: *Oryza sativa* (Rice) and *Zea mays* (Maize). *Chlorella* sp. was used to root the tree (see Table S2, Additional file 8). Proteins sequences were acquired from the NCBI database (http://ncbi.nlm.nih.gov). Alignment was performed with ClustalX software [64]. The phylogenic tree was inferred with Neighborhood Joining (NJ) [65], bootstrap 1000 and Maximum Likelihood method based on Equal input model [66], bootstrap 100, by using MEGA6 software [67].

### Chromosome location comparison of kelch motif *At*ACBPs, *Br*ACBPs, *Bo*ACBPs and *Bn*ACBPs

Comparison in chromosome location was based on kelch motif ACBPs. Hypothetical chromosome location of each *Bn*ACBPs was obtained from CNS-Genoscope database (http://www.genoscope.cns.fr/brassicanapus/). Chromosome locations of *At*ACBPs, *Br*ACBPs and *Bo*ACBPs were obtained from TAIR (http://www.arabidopsis.org) and from BRAD (http://brassicadb.org). In order to know the arrangement of *B. napus* genes in the chromosome, genes neighbors of kelch motif *Bn*ACBPs were searched and associated to their respective homologues in *B. rapa, B. oleracea* and *A. thaliana.*

## Availability of supporting data

The data set of Fig. 2 supporting the results of this article is available in the TreeBase (http://treebase.org/treebase-web/home.html) repository, under the accession URL http://purl.org/phylo/treebase/phylows/study/TB2:S17727.

## Additional files

Additional file 1: Figure S1. Alignment of kelch domains in *At*ACBPs and *Bn*ACBPs. I, II, III, IV and V are the order of kelch domain on ACBPs. Highly conserved residues are in yellow, identical residues are in blue. Red framed residues (II) are putative altered residues. Additional file 2: Figure S2. Alignment of the four classes of *At*ACBPs and *Bn*ACBPs. ACBD are framed in yellow, conserved residues are noticed. Ankyrin domains are framed in green, and kelch domain in blue. Additional file 3: Figure S3. Comparison map of kelch motif *At*ACBP, *Br*ACBP, *Bo*ACBP and *Bn*ACBP: AIS76196. Additional file 4: Figure S4. Comparison map of kelch motif *At*ACBP, *Br*ACBP, *Bo*ACBP and *Bn*ACBP: AIS76195. Additional file 5: Figure S5. Comparison map of kelch motif *At*ACBP, *Br*ACBP, *Bo*ACBP and *Bn*ACBP: AIS76197 and AIS76198. Additional file 6: Figure S6. Evolutionary history of the *Brassica* genomes, based on ACBPs. Homology relationships are illustrated for *B. rapa* (AA), *B. oleracea* (CC) and *B. napus* (AACC). Chromosome location are mentioned. Underlined genes were lost during evolution. The tree was build with NJ methods and MEGA 6. Additional file 7: Table S1. Primer sequences used to clone kelch motif *Bn*ACBPs. Additional file 8: Table S2. List of plants species used in phylogenetic analysis.
