# Supplementary figures and images for: Genome-wide identification and Phylogenic analysis of kelch motif containing ACBP in Brassica napus
Source: BMC Genomics. 2015 Jul 9;16(1):512. doi: 10.1186/s12864-015-1735-6 (PMC4497377; doi:10.1186/s12864-015-1735-6)

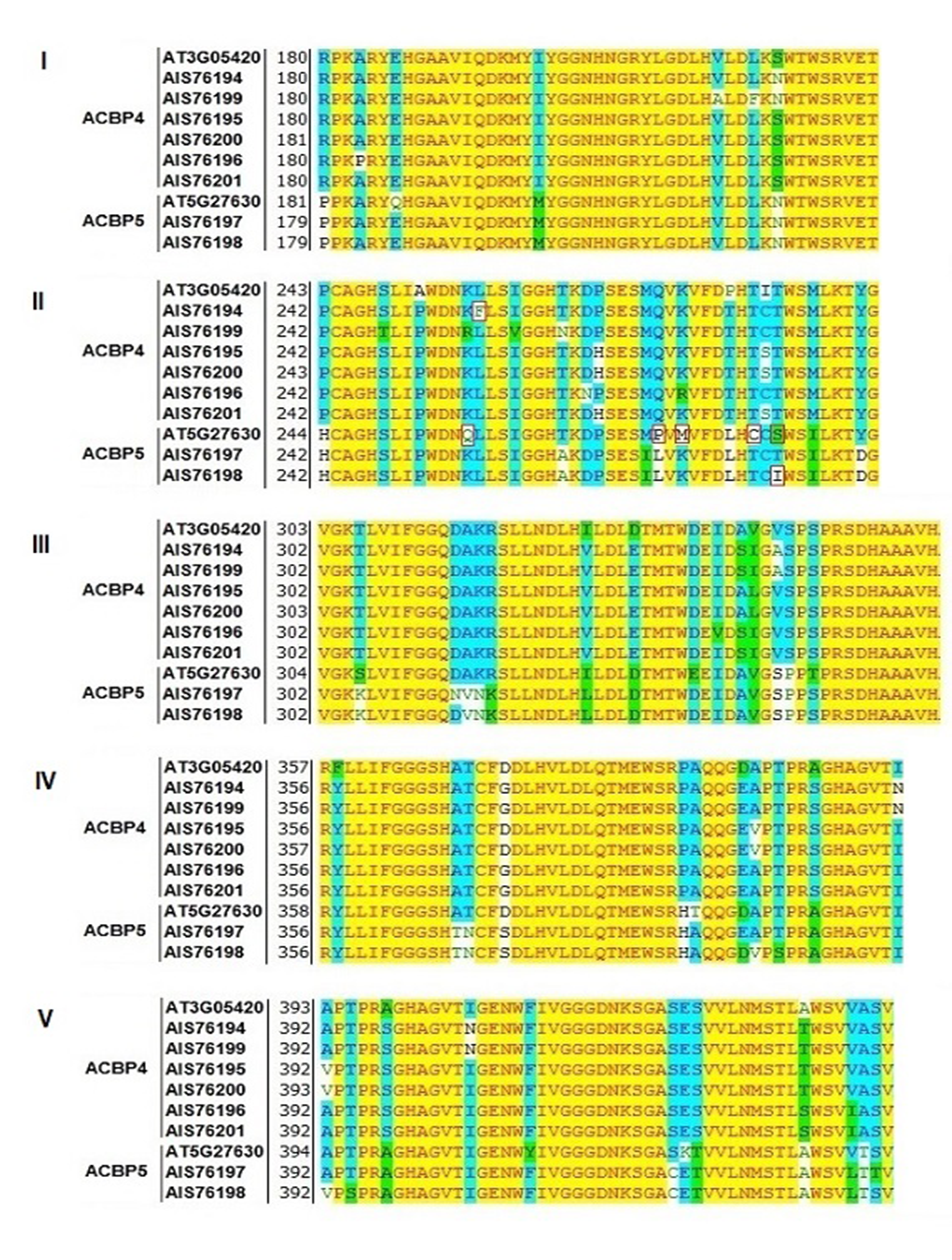

Supplement: Additional file 1: Figure S1. — Alignment of kelch domains in AtACBPs and BnACBPs. I, II, III, IV and V are the order of kelch domain on ACBPs. Highly conserved residues are in yellow, identical residues are in blue. Red framed residues (II) are putative altered residues. [file 12864_2015_1735_MOESM1_ESM.tiff]

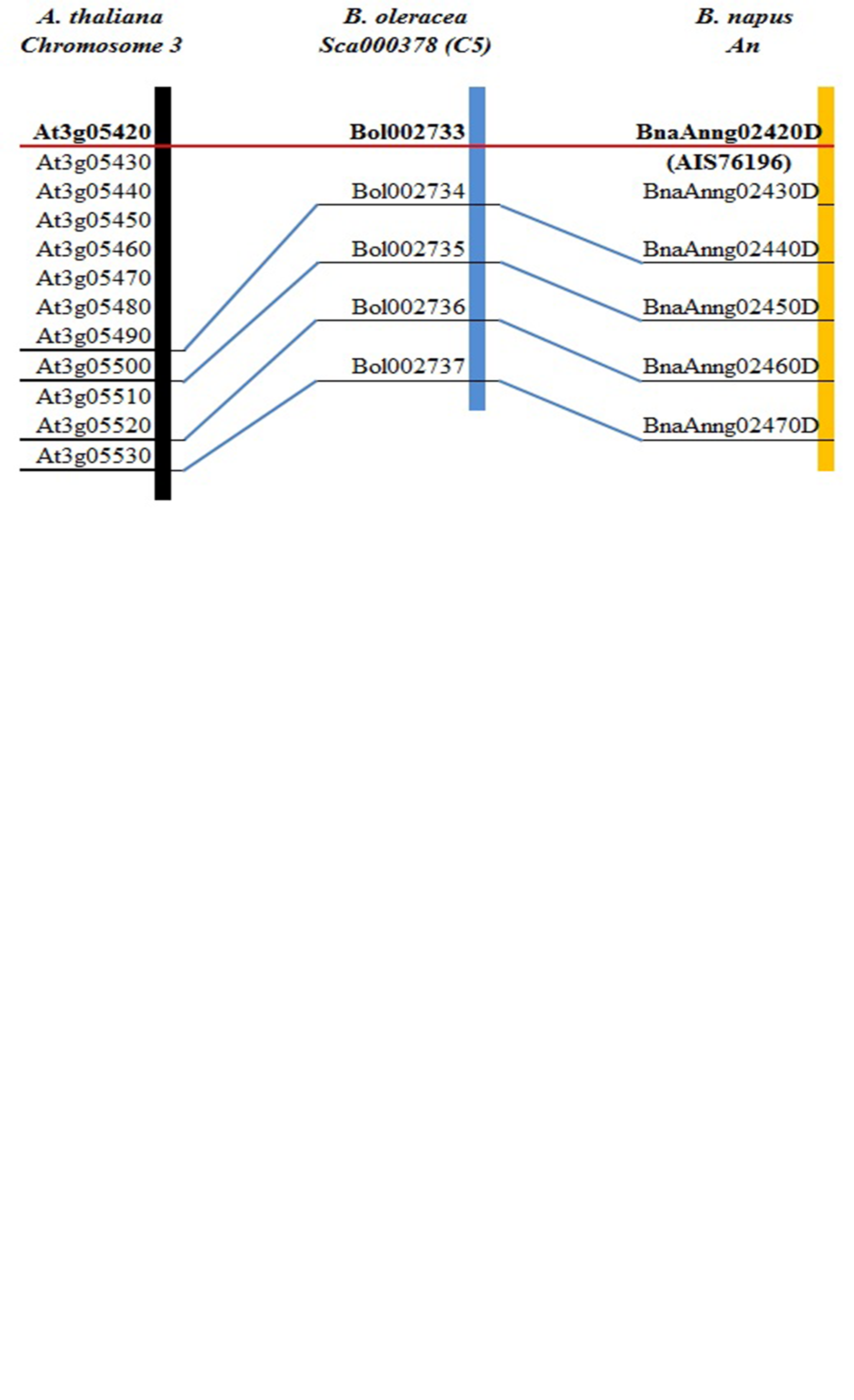

Supplement: Additional file 3: Figure S3. — Comparison map of kelch motif AtACBP, BrACBP, BoACBP and BnACBP: AIS76196. [file 12864_2015_1735_MOESM3_ESM.tiff]

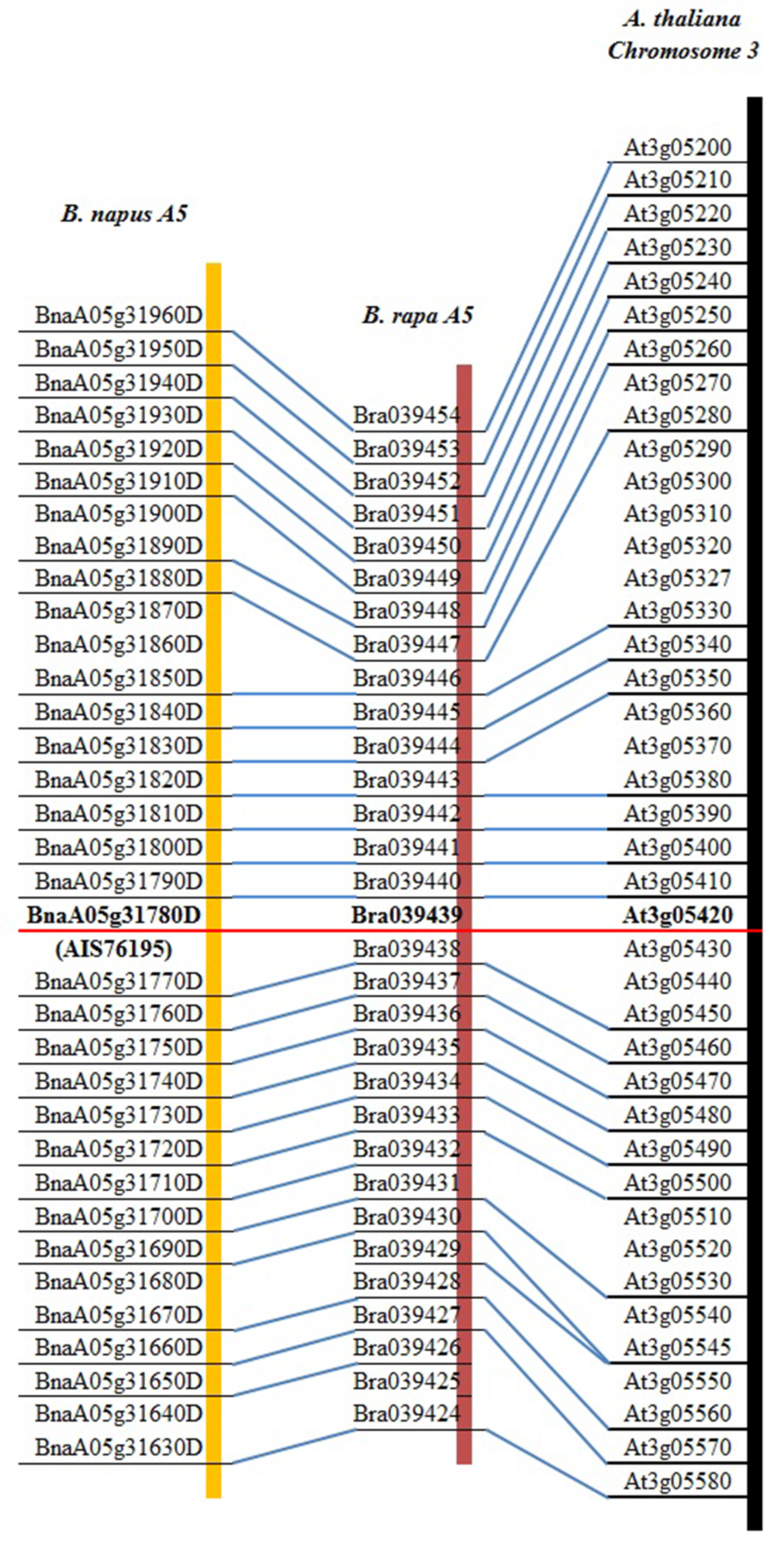

Supplement: Additional file 4: Figure S4. — Comparison map of kelch motif AtACBP, BrACBP, BoACBP and BnACBP: AIS76195. [file 12864_2015_1735_MOESM4_ESM.tiff]

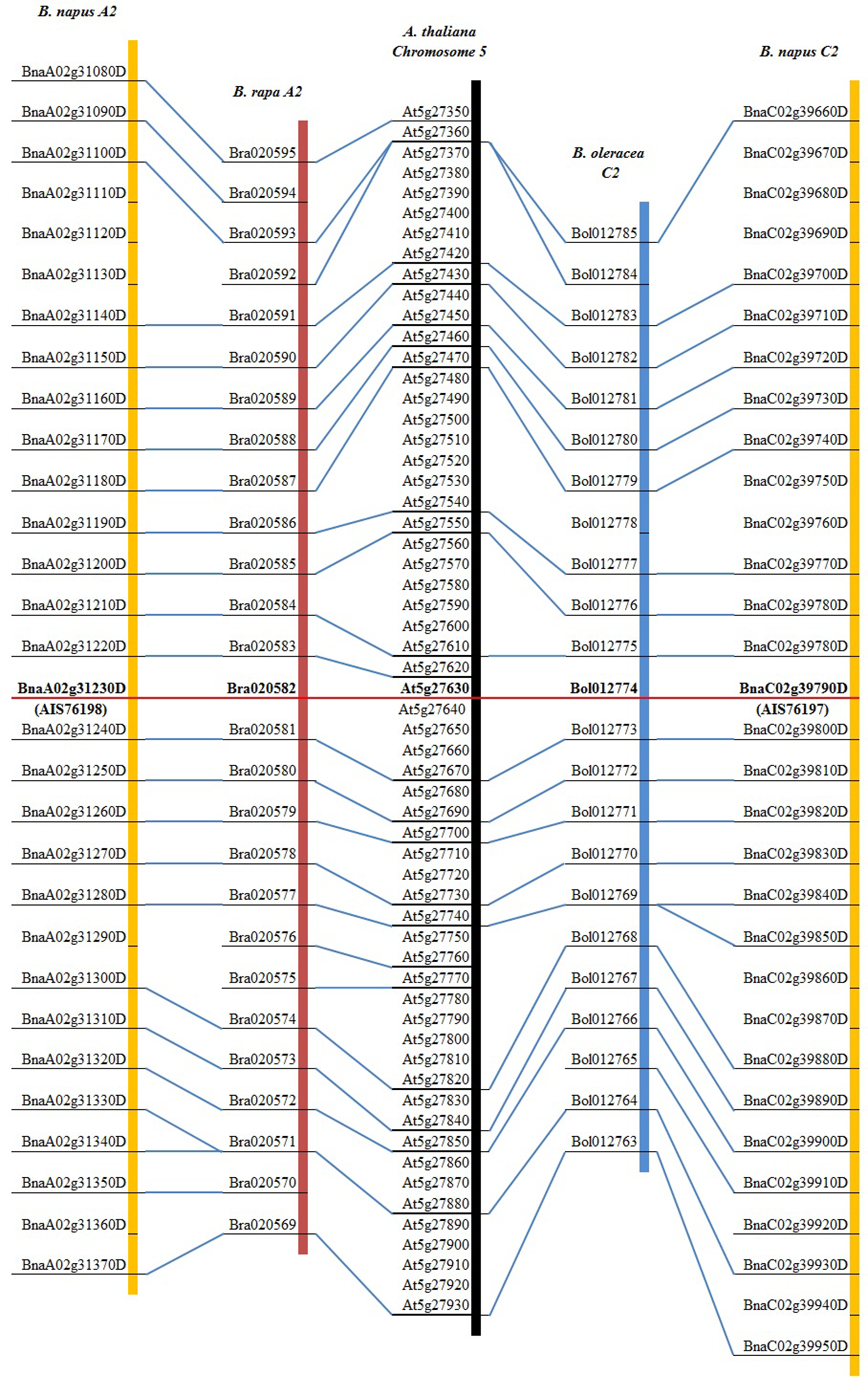

Supplement: Additional file 5: Figure S5. — Comparison map of kelch motif AtACBP, BrACBP, BoACBP and BnACBP: AIS76197 and AIS76198. [file 12864_2015_1735_MOESM5_ESM.tiff]

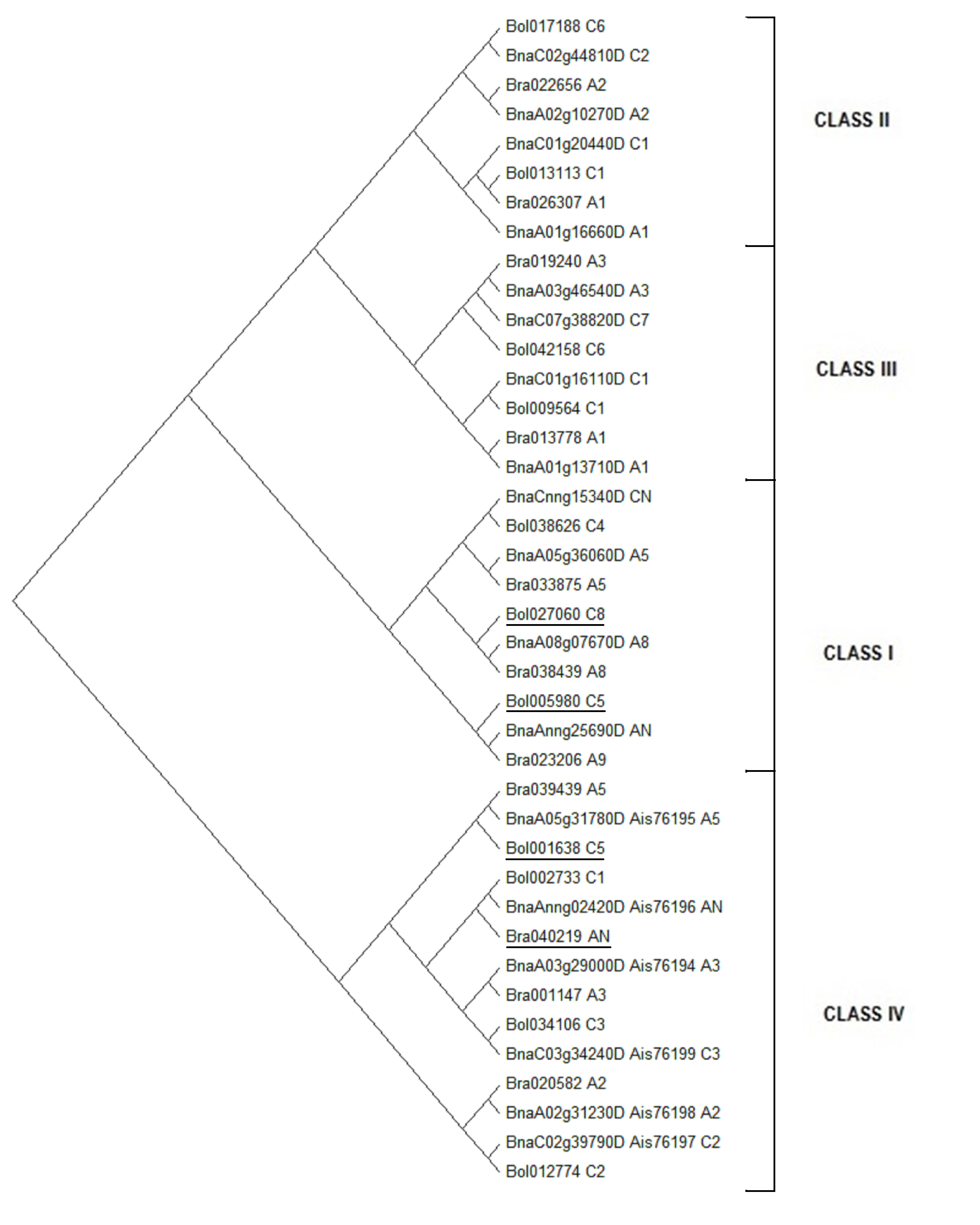

Supplement: Additional file 6: Figure S6. — Evolutionary history of the Brassica genomes, based on ACBPs. Homology relationships are illustrated for B. rapa (AA), B. oleracea (CC) and B. napus (AACC). Chromosome location are mentioned. Underlined genes were lost during evolution. The tree was build with NJ methods and MEGA 6. [file 12864_2015_1735_MOESM6_ESM.tiff]
