# Supplementary material for: Genome-wide identification and Phylogenic analysis of kelch motif containing ACBP in Brassica napus
Source: BMC Genomics. 2015 Jul 9;16(1):512. doi: 10.1186/s12864-015-1735-6 (PMC4497377; doi:10.1186/s12864-015-1735-6)
Supplement: Additional file 2: Figure S2. — Alignment of the four classes of AtACBPs and BnACBPs. ACBD are framed in yellow, conserved residues are noticed. Ankyrin domains are framed in green, and kelch domain in blue. [file 12864_2015_1735_MOESM2_ESM.pdf]

```
PileUp

   MSF: 923   Type: P   Check: 153   ..

 Name: AT1G31812_ClassI         Len: 923        Check: 5687     Weight: 0
 Name: BNAANNG25690D_ClassI     Len: 923        Check: 5539     Weight: 0
 Name: BNAA05G36060D_ClassI     Len: 923        Check: 6046     Weight: 0
 Name: BNAA08G07670D_ClassI     Len: 923        Check: 6926     Weight: 0
 Name: BNACNNG15340D_ClassI     Len: 923        Check: 6442     Weight: 0
 Name: AT5G53470_ClassII        Len: 923        Check: 3043     Weight: 0
 Name: BNAA02G10270D_ClassII    Len: 923        Check: 3730     Weight: 0
 Name: BNAC02G44810D_ClassII    Len: 923        Check: 1387     Weight: 0
 Name: AT4G27780_ClassII        Len: 923        Check: 5422     Weight: 0
 Name: BNAC01G20440D_ClassII    Len: 923        Check: 2404     Weight: 0
 Name: BNAA01G16660D_ClassII    Len: 923        Check: 2616     Weight: 0
 Name: AT4G24230_ClassIII       Len: 923        Check: 9729     Weight: 0
 Name: BNAA01G13710D_ClassIII   Len: 923        Check: 7116     Weight: 0
 Name: BNAC01G16110D_ClassIII   Len: 923        Check: 860      Weight: 0
 Name: BNAA03G46540D_ClassIII   Len: 923        Check: 3252     Weight: 0
 Name: BNAC07G38820D_ClassIII   Len: 923        Check: 7934     Weight: 0
 Name: AT3G05420_ClassIV        Len: 923        Check: 2608     Weight: 0
 Name: AIS76194_ClassIV         Len: 923        Check: 5777     Weight: 0
 Name: AIS76199_ClassIV         Len: 923        Check: 5917     Weight: 0
 Name: AIS76196_ClassIV         Len: 923        Check: 3471     Weight: 0
 Name: AIS76201_ClassIV         Len: 923        Check: 1746     Weight: 0
 Name: AIS76195_ClassIV         Len: 923        Check: 5264     Weight: 0
 Name: AIS76200_ClassIV         Len: 923        Check: 6072     Weight: 0
 Name: AT5G27630_ClassIV        Len: 923        Check: 4259     Weight: 0
 Name: AIS76197_ClassIV         Len: 923        Check: 8774     Weight: 0
 Name: AIS76198_ClassIV         Len: 923        Check: 8132     Weight: 0

//

                        1                                                50
      AT1G31812_ClassI  .......... .......... .......... .......... ..........
  BNAANNG25690D_ClassI  .......... .......... .......... .......... ..........
  BNAA05G36060D_ClassI  .......... .......... .......... .......... ..........
  BNAA08G07670D_ClassI  .......... .......... .......... .......... ..........
  BNACNNG15340D_ClassI  .......... .......... .......... .......... ..........
     AT5G53470_ClassII  .......... .......... .......... .......... ..........
 BNAA02G10270D_ClassII  .......... .......... .......... .......... ..........
 BNAC02G44810D_ClassII  .......... .......... .......... .......... ..........
     AT4G27780_ClassII  .......... .......... .......... .......... ..........
 BNAC01G20440D_ClassII  .......... .......... .......... .......... ..........
 BNAA01G16660D_ClassII  .......... .......... .......... .......... ..........
    AT4G24230_ClassIII  MEVFLEMLLT AVVALLFSFL LAKLVSVATV .ENDLSS.DQ PLKPEIGVG.
BNAA01G13710D_ClassIII  MEFLLELLLT AVVALLFSFL VAKIVSVSVA GENDGSS.DQ AGETEIGVGD
BNAC01G16110D_ClassIII  MEFLLELLLT AVVALLFSFL VAKIVSVSMA GES.....DR IEKTEIGVGD
BNAA03G46540D_ClassIII  MEFFLEMLLT AVVAVLFSFL VAKLVSVSMV GNSGGVVNDQ AEETEIGVV.
BNAC07G38820D_ClassIII  MEFFLEMLLT AVVAVLFSFL VAKLVSVPMA GSSGGVVNDQ AEENEIGVV.
     AT3G05420_ClassIV  .......... .......... .......... .......... ..........
      AIS76194_ClassIV  .......... .......... .......... .......... ..........
      AIS76199_ClassIV  .......... .......... .......... .......... ..........
      AIS76196_ClassIV  .......... .......... .......... .......... ..........
      AIS76201_ClassIV  .......... .......... .......... .......... ..........
      AIS76195_ClassIV  .......... .......... .......... .......... ..........
      AIS76200_ClassIV  .......... .......... .......... .......... ..........
```

```
         AT5G27630_ClassIV  .......... .......... .......... .......... ..........
          AIS76197_ClassIV  .......... .......... .......... .......... ..........
          AIS76198_ClassIV  .......... .......... .......... .......... ..........

                            51                                                100
           AT1G31812_ClassI  .......... .......... .......... .......... ..........
     BNAANNG25690D_ClassI  .......... .......... .......... .......... ..........
     BNAA05G36060D_ClassI  .......... .......... .......... .......... ..........
     BNAA08G07670D_ClassI  .......... .......... .......... .......... ..........
     BNACNNG15340D_ClassI  .......... .......... .......... .......... ..........
        AT5G53470_ClassII  .......... .......... .......... .......... ..........
    BNAA02G10270D_ClassII  .......... .......... .......... .......... ..........
    BNAC02G44810D_ClassII  .......... .......... .......... .......... ..........
        AT4G27780_ClassII  .......... .......... .......... .......... ..........
    BNAC01G20440D_ClassII  .......... .......... .......... .......... ..........
    BNAA01G16660D_ClassII  .......... .......... .......... .......... ..........
      AT4G24230_ClassIII  ....VTE.DV RFGMKMDARV LESQR..NFQ VVDENVELVD RFL.SEEADR
  BNAA01G13710D_ClassIII  GSATVEE..L CFGLKVDAPV VQSERKLRA. VVDENVEHVD RFGS.GADRV
  BNAC01G16110D_ClassIII  GSATVEE..L CFGLKVDAPV VQSERKLR.V VVDEN...VD RFG.NGADRV
  BNAA03G46540D_ClassIII  ..A.VEEE.L CSGLKVDAPV VQSERRLGAV VVDENVERVD RFG.SEADRV
  BNAC07G38820D_ClassIII  ..A.VEEE.L CSGLKMDAPV VQSQRRLGVA VVDENVERVD RFG.SEADRV
        AT3G05420_ClassIV  .......... .......... .......... .......... ..........
         AIS76194_ClassIV  .......... .......... .......... .......... ..........
         AIS76199_ClassIV  .......... .......... .......... .......... ..........
         AIS76196_ClassIV  .......... .......... .......... .......... ..........
         AIS76201_ClassIV  .......... .......... .......... .......... ..........
         AIS76195_ClassIV  .......... .......... .......... .......... ..........
         AIS76200_ClassIV  .......... .......... .......... .......... ..........
        AT5G27630_ClassIV  .......... .......... .......... .......... ..........
         AIS76197_ClassIV  .......... .......... .......... .......... ..........
         AIS76198_ClassIV  .......... .......... .......... .......... ..........

                            101                                               150
           AT1G31812_ClassI  .......... .......... .......... .......... ..........
     BNAANNG25690D_ClassI  .......... .......... .......... .......... ..........
     BNAA05G36060D_ClassI  .......... .......... .......... .......... ..........
     BNAA08G07670D_ClassI  .......... .......... .......... .......... ..........
     BNACNNG15340D_ClassI  .......... .......... .......... .......... ..........
        AT5G53470_ClassII  .......... .......... .......... .......... ..........
    BNAA02G10270D_ClassII  .......... .......... .......... .......... ..........
    BNAC02G44810D_ClassII  .......... .......... .......... .......... ..........
        AT4G27780_ClassII  .......... .......... .......... .......... ..........
    BNAC01G20440D_ClassII  .......... .......... .......... .......... ..........
    BNAA01G16660D_ClassII  .......... .......... .......... .......... ..........
      AT4G24230_ClassIII  VYEVDEAVTG NAKICGDREA E....SSAAA SSENYVIAEE VILVRGQDEQ
  BNAA01G13710D_ClassIII  ...VDEVEEA ARDVELVVLT AEADELLAAV SPGNVVAKEM IVRGEEETGY
  BNAC01G16110D_ClassIII  ...VDEVEEA ARDVELVVLT TEANEFLAAV SPGNVIAKEM IVRGEEETGD
  BNAA03G46540D_ClassIII  ...VDEVKEG TKGEDWVVTS DE...SSAAG SPENVLAEEM MVCGEDKQRD
  BNAC07G38820D_ClassIII  ...VDEFEEA GEGEDLVVTS DE...SSAAV SPENVIAEEM MARGEDKQRD
        AT3G05420_ClassIV  .......... .......... .......... .......... ..........
         AIS76194_ClassIV  .......... .......... .......... .......... ..........
         AIS76199_ClassIV  .......... .......... .......... .......... ..........
         AIS76196_ClassIV  .......... .......... .......... .......... ..........
         AIS76201_ClassIV  .......... .......... .......... .......... ..........
         AIS76195_ClassIV  .......... .......... .......... .......... ..........
         AIS76200_ClassIV  .......... .......... .......... .......... ..........
        AT5G27630_ClassIV  .......... .......... .......... .......... ..........
```

```
      AIS76197_ClassIV  .......... .......... .......... .......... ..........
      AIS76198_ClassIV  .......... .......... .......... .......... ..........

                        151                                                   200
      AT1G31812_ClassI  .......... .......... .......... .......... ..........
  BNAANNG25690D_ClassI  .......... .......... .......... .......... ..........
  BNAA05G36060D_ClassI  .......... .......... .......... .......... ..........
  BNAA08G07670D_ClassI  .......... .......... .......... .......... ..........
  BNACNNG15340D_ClassI  .......... .......... .......... .......... ..........
     AT5G53470_ClassII  ......MADW YQLAQSIIFG LIFAYLLAKL ISILLAFKDE NLSLTRNHTT
 BNAA02G10270D_ClassII  .....MGVDW FQLAQSLIFG LIFAYLLAKL ISILVAFKDD NLSLTRSHDA
 BNAC02G44810D_ClassII  .....MGGDW YHLAQSLIFG LIFAYLLAKL ISILVAFKDD NLSLTRSHDA
     AT4G27780_ClassII  ......MGDW AQLAQSVILG LIFSYLLAKL ISIVVTFKED NLSLTRHPEE
 BNAC01G20440D_ClassII  ......MGDW AQLAQSVIIG LIFSYLLAKL ISIVVTFKED NLSLTRHHDP
 BNAA01G16660D_ClassII  ......MGDW AQLAQSVIIG LIFSYLLAKL ISIVVTFKED NLSLTRHHDP
    AT4G24230_ClassIII  SDS......A EAESISSVSP ENVVAEEIKS QGQEEVTELG RSGCVENEES
BNAA01G13710D_ClassIII  EREELVVSTA EAESTASISP ENVITEEIMN RGQEEGTE.G RSDCVEN..V
BNAC01G16110D_ClassIII  ERQELIESTA EAESTASVVQ ENMIAEEIIN RGHEEETE.G VSSCVE....
BNAA03G46540D_ClassIII  AAEEFNVRTV GAESTASVSL ENVRAEEIMI DG.EEVRS.E ED.VISGE..
BNAC07G38820D_ClassIII  AAEEFNVRTV GAESTASVSL ENVRAEEIMI GG.EKVRS.E ED.VISGE..
      AT3G05420_ClassIV  .......... .......... .......... .......... ..........
      AIS76194_ClassIV  .......... .......... .......... .......... ..........
      AIS76199_ClassIV  .......... .......... .......... .......... ..........
      AIS76196_ClassIV  .......... .......... .......... .......... ..........
      AIS76201_ClassIV  .......... .......... .......... .......... ..........
      AIS76195_ClassIV  .......... .......... .......... .......... ..........
      AIS76200_ClassIV  .......... .......... .......... .......... ..........
     AT5G27630_ClassIV  .......... .......... .......... .......... ..........
      AIS76197_ClassIV  .......... .......... .......... .......... ..........
      AIS76198_ClassIV  .......... .......... .......... .......... ..........

                        201                                                   250
      AT1G31812_ClassI  .......... .......... .......... .......... ..........
  BNAANNG25690D_ClassI  .......... .......... .......... .......... ..........
  BNAA05G36060D_ClassI  .......... .......... .......... .......... ..........
  BNAA08G07670D_ClassI  .......... .......... .......... .......... ..........
  BNACNNG15340D_ClassI  .......... .......... .......... .......... ..........
     AT5G53470_ClassII  Q....SEYEN L..RKVETLT GIS.GETDSL IAEQGSLRGD EDESDDDD..
 BNAA02G10270D_ClassII  AR...S..EN EYYRKVDSSA ....GETDSL VAEQGSLRGD E...DDDD.E
 BNAC02G44810D_ClassII  Q....S..EN DYHRKVDSST ....GETDSL VAEQGSLRGD E...DDDD..
     AT4G27780_ClassII  SQLEIKPEGV DSRRLDSSCG GFGGEADSLV AEQGSSRSDS VAGDDSEEDD
 BNAC01G20440D_ClassII  EPELKNLKPE VDSRRIESST G....EADSL VAEQGSSRGD SVAGDTEDED
 BNAA01G16660D_ClassII  EPESKNLKPE VDSRRIESST G....EADSL VAEQGSSRGD SVAGDTEEDD
    AT4G24230_ClassIII  GGDVLVAESE EVR......V EKSSNMVEES DAEAENEEKT ELTIEE...D
BNAA01G13710D_ClassIII  KREVVVTESE KVRVEESNSV EKSEDKMELS IEEQVELSIE EEDL.....D
BNAC01G16110D_ClassIII  RGEVVVTESE EVRVEESNSG EKSEDKMEFC IEEQVELSIE EDDD.....D
BNAA03G46540D_ClassIII  ...LVVAESE DVRVEESNTV EESENKMEFN TKEGDKEEKK ELSIEEEE.D
BNAC07G38820D_ClassIII  ...VVVAEPE DVRVEESNTV EECEHKMELD TIGEDKE.KE ELSIEEED.D
      AT3G05420_ClassIV  .......... .......... .......... .......... .........M
      AIS76194_ClassIV  .......... .......... .......... .......... .........M
      AIS76199_ClassIV  .......... .......... .......... .......... .........M
      AIS76196_ClassIV  .......... .......... .......... .......... .........M
      AIS76201_ClassIV  .......... .......... .......... .......... .........M
      AIS76195_ClassIV  .......... .......... .......... .......... .........M
      AIS76200_ClassIV  .......... .......... .......... .......... .........M
     AT5G27630_ClassIV  .......... .......... .......... .......... ........MA
      AIS76197_ClassIV  .......... .......... .......... .......... ..........
```

```
        AIS76198_ClassIV  .......... .......... .......... .......... ..........

                          251                                              300
        AT1G31812_ClassI  ........MG LKEEFEEHAE KVNTLTEL.. ........PS NEDLLILYGL
    BNAANNG25690D_ClassI  ........MG LKGEFEEHAE KVKTLTTK.. ........PS DEDLLILYGL
    BNAA05G36060D_ClassI  ........MG LKEDFEEHAE KVKKLTAS.. ........PS NEDLLILYGL
    BNAA08G07670D_ClassI  ........MG LKEDFEEHAE NVKKLTTS.. ........PS NEDLLILYGL
    BNACNNG15340D_ClassI  ........MG LKEEFEEQAE KVKKLTAS.. ........PS NEDLLILYGL
       AT5G53470_ClassII  ..WEGVESTE LDEAFSAATA FVAAAASD.. ...RLSQKVS NELQLQLYGL
   BNAA02G10270D_ClassII  EDWEGVESTE LDEAFSAATA FVAAAASD.. ...RLSQKVS SELQLQLYGL
   BNAC02G44810D_ClassII  .DWEGVESTE LDEAFSAATA FVAAAASD.. ...RLSQKVS NELQLQLYGL
       AT4G27780_ClassII  .DWEGVESTE LDEAFSAATL FVTTAAAD.. ...RLSQKVP SDVQQQLYGL
   BNAC01G20440D_ClassII  DDWEGVESTE LDEAFSAATL FVTTAASD.. ...RLSQKVP SEVQQQLYGL
   BNAA01G16660D_ClassII  DDWEGVESTE LDEAFSAATL FVTTAASD.. ...RLSQKVP SEVLQQLYGL
      AT4G24230_ClassIII  DDWEGIERSE LEKAFAAAVN LLEESGKA.. ......EEIG AEAKMELFGL
  BNAA01G13710D_ClassIII  DDWEGIERSE LEIAFAAASN LLEESGKG.. ......EDIG AEAKMELYGL
  BNAC01G16110D_ClassIII  DDWEGIEKSE LEITFSAASN LLEQSGKG.. ......EEIT AEAKMELYGL
  BNAA03G46540D_ClassIII  DDWEGIERSE LEKAFAATSS LLEVSGKA.. ......EEVG DEVKMELYGL
  BNAC07G38820D_ClassIII  DDWEGIERSE LEKAFAATSS LLEESGKA.. ......EEIG DEVKMELYGL
       AT3G05420_ClassIV  AMPRATSGPA YPERFYAAAS YVGLDGSD.S SAKNVISKFP DDTALLLYAL
        AIS76194_ClassIV  AITRATSGPA YPERFYAAAS YVGLDGSD.S SAKHVSSKFS NDTALIFYAL
        AIS76199_ClassIV  AITRATSGPA YPERFYAAAS YVGLDGSD.S SAKHVSSKFS NDTALIFYAL
        AIS76196_ClassIV  AMARATSGPA YPERFYAAAS YVGFDGSD.S SAKHVSSKFS DDTSLILYAL
        AIS76201_ClassIV  AMARATSGPA YPERFYAAAS YAGFDGSE.S SAKNVSSKFS NDTALLLYAL
        AIS76195_ClassIV  AMARATSGPA YPERFYAAAS YAGFDGSE.S SAKNVSSKFS NDTALLLYAL
        AIS76200_ClassIV  AMARATSGPA YPERFYAAAS YAGFDGSE.S SAKNVSSKFS NDTALPLYAL
       AT5G27630_ClassIV  HMVRASSGLS YPERFYAAAS YVGLDGSQ.S SVKQLSSKFS NDTSLLLYTL
        AIS76197_ClassIV  .MAKQSATLA YPDRFYAAAS YLGLDGSAPS SVKQLSSKFS NDTALLLYAL
        AIS76198_ClassIV  .MAKASATLA YPDRFYAAAS YLGLDGSVPS SVKQLSSKFS NDTALLLHAL

                          301                                              350
        AT1G31812_ClassI  YKQAKFGPVD TSRPGMFSMK ERAKWDAWKA VEGKSSEEAM NDYITKVKQL
    BNAANNG25690D_ClassI  FKQATVGPVT TSRPGMFSMK ERAKWDAWKA VEAKSTDEAM SDYITKVKQL
    BNAA05G36060D_ClassI  YKQATVGPVT TSRPGMFSMK ERAKWDAWKA VEGKSTDEAM SDYITKVKQL
    BNAA08G07670D_ClassI  YKQATVGPVT TSRPGMFSMK ERAKWDAWKA VEGKSTDEAM SDYITKVKQL
    BNACNNG15340D_ClassI  YKQATVGPVT TSRPGMFSMK ERAKWDAWKA VEGKSTDEAM SDYITKVKQL
       AT5G53470_ClassII  YKIATEGPCT APQPSALKMT ARAKWQAWQK LGAMPPEEAM EKYIDLVTQL
   BNAA02G10270D_ClassII  YKIATEGACT APQPSALKMT ARAKWQAWQK MGAMPAEEAM EKYIDLVTHL
   BNAC02G44810D_ClassII  YKIATEGACT APQPSALKMT ARAKWQAWHK LGAMPAEEAM EKYIDLVTHL
       AT4G27780_ClassII  YKIATEGPCT APQPSALKMT ARAKWQAWQK LGAMPPEEAM EKYIEIVTQL
   BNAC01G20440D_ClassII  YKIATEGPCT APQPSALKIT ARAKWQAWQK LGAMPPEEAM EKYIEIVTQL
   BNAA01G16660D_ClassII  YKIATEGPCT APQPSALKIT ARAKWQAWQK LGAMPPEEAM EKYIEIVTQL
      AT4G24230_ClassIII  HKIATEGSCR EAQPMAVMIS ARAKWNAWQK LGNMSQEEAM EQYLALVSKE
  BNAA01G13710D_ClassIII  HKIATEGSCR EAQPMAVMLS ARAKWNAWQR LGNMSQEEAM EQYLALVSKE
  BNAC01G16110D_ClassIII  HKIATEGSCR EAQPMAIMLS ARAKWNAWQR LGNMSQEEAM EQYLALVSKE
  BNAA03G46540D_ClassIII  YMIATEGSCR ETQPMAIMVS ARAKWNAWQK LGNMSQEEAM EKYLALVSKE
  BNAC07G38820D_ClassIII  YKIATEGSCR ETQPMAIMVS ARAKWNAWQK LGNMSQEEAM EKYLALVSKE
       AT3G05420_ClassIV  YQQATVGPCN TPKPSAWRPV EQSKWKSWQG LGTMPSIEAM RLFVKILEED
        AIS76194_ClassIV  YQQATVGPCS TPKPSAWRPV EKSKWKSWQG LGTMPSIEAM RLFVKILEEE
        AIS76199_ClassIV  YQQATVGPCN TPKPSAWRPV EQSKWKSWQG LGTMPSIEAM RLFVKILEEE
        AIS76196_ClassIV  YQQATVGPCN TPKPSAWRPV EQSKWQSWQG LGTMPSIEAM RLFVKILEEE
        AIS76201_ClassIV  YQQATVGPCN TPKPSAWRPV EQSKWQSWQG LGTMPSIEAM RLFVKILEEE
        AIS76195_ClassIV  YQQATVGPCN TPKPSAWRPV EQSKWKSWQG LGTMPSMEAM RLFVKILEED
        AIS76200_ClassIV  YQQATVGPCN TPKPSAWRPV EQSKWRSWQG LGTMPSMEAM RLFVKILEED
       AT5G27630_ClassIV  HQQATLGPCS IPKPSAWNPV EQSKWKSWQG LGTMPSIEAM RLFVKILEEA
        AIS76197_ClassIV  HQQATVGPCN TPKPSAWNPA EQSKWKSWQG LGTMPSIEAK RHFVKILEEG
        AIS76198_ClassIV  HKQTTVGPCN VPKPSAWNPA EQSKWRSWQG LGTMPSIDAM RHFVKILEEG
```

```
                        351                                                400
      AT1G31812_ClassI  LEVAASKAST .......... .......... .......... ..........
  BNAANNG25690D_ClassI  MEAAEAST.. .......... .......... .......... ..........
  BNAA05G36060D_ClassI  LEAEASSASA .......... .......... .......... ..........
  BNAA08G07670D_ClassI  LEAEAAAAST .......... .......... .......... ..........
  BNACNNG15340D_ClassI  LEAEASSASA .......... .......... .......... ..........
     AT5G53470_ClassII  YPAWVEGGSK RRNRSGEAAG .....PMGPV FSSLVYEEES DNEL......
 BNAA02G10270D_ClassII  YPAWVEGGSK GRNRSGDAAG SSSRAPMGPV FSSLVYEEES ENEL......
 BNAC02G44810D_ClassII  YPAWVEGGSK GSNRSGDAAG SNSRAPMGPV FSSLVYEEES ENDL......
     AT4G27780_ClassII  YPTWLDGGVK AGSRGGDDAA SNSRGTMGPV FSSLVYDEES ENEL......
 BNAC01G20440D_ClassII  YPTWLDGGVK AGSGSKDDAV SNTGGTMGPV FSSLVYEEES ENELHSSMRS
 BNAA01G16660D_ClassII  YPTWLDGGVK AGSGSKDEAV SNTGGTMGPV FSSLVYEEES EIELHSSMRS
    AT4G24230_ClassIII  IPGLTKAGHT VGKMSEMETS VGLPPNSGSL EDPTNLVTTG VDESSKN...
BNAA01G13710D_ClassIII  IPDLVN...T VGKMPETETS VDLPPNSGSL EDPTTLDTIG VAT.SKNEIH
BNAC01G16110D_ClassIII  IPGLLN...T VGKMPETETS VDLR....SL EDPTTLDTIG FPT.SKN...
BNAA03G46540D_ClassIII  IPGLMN...T VGKIPV.... ..LPPNSGSL EDPTTLGTTG VAF.SKN...
BNAC07G38820D_ClassIII  IPGLMN...T VGKIPV.... ..LPPNPGSL EDPTTLGTTG VAF.SKN...
     AT3G05420_ClassIV  DPGWYSRASN DIPDPVVDVQ IN.RAKDEPV VENGSTFSET KTISTENGRL
      AIS76194_ClassIV  NPDWYSREFT DIPDPVVDVQ IN.QTKDEPV VENGNSLSET KTVSAENGRL
      AIS76199_ClassIV  NPDWYSSEFT DIPDPVVDVQ IN.QTKDEPV VENGNSLSET KTISAENGRL
      AIS76196_ClassIV  NPSWYSRASN DIPDPVIDVQ IN.TTKEEPV VENGNSFSET KTVSAENGHL
      AIS76201_ClassIV  NPSWYSRASN DIPDPVIDVQ IN.TTKDEPV VENGNSFSET KTISAENGGL
      AIS76195_ClassIV  DPTWYSRASK DIPDPVVDVQ IN.TTKDEPV VENGNSFSET KTISAENGGL
      AIS76200_ClassIV  DPTWYSRASK DIPDPVVDVQ INQATKDEPV VENGNSFSET KTISAENGGL
     AT5G27630_ClassIV  DPGWYPRTSN SVLDPAVHVQ IN.STKAEPS FESGASFGET KTITSEDGRL
      AIS76197_ClassIV  DSSWYPNPPN SVPDPAIDVQ IS.STKAEPS VENGGSFGET MTTATEDGRL
      AIS76198_ClassIV  DPSWYPNPPN SAPEPAVDFQ IS.STKAEAS VENGGSFGET MTTATEDGRL

                        401                                                450
      AT1G31812_ClassI  .......... .......... .......... .......... ..........
  BNAANNG25690D_ClassI  .......... .......... .......... .......... ..........
  BNAA05G36060D_ClassI  .......... .......... .......... .......... ..........
  BNAA08G07670D_ClassI  .......... .......... .......... .......... ..........
  BNACNNG15340D_ClassI  .......... .......... .......... .......... ..........
     AT5G53470_ClassII  .......KID AIHAFAREGE VENLLKCIEN GIPVNARDSE GRTPLHWAID
 BNAA02G10270D_ClassII  .......KID AVHAFAREGE VENLLKCIES GIPVNARDSE GRTPLHWAID
 BNAC02G44810D_ClassII  .......KID AVHAFAREGE VENLLKCIES GIPVNARDSE GRTPLHWAID
     AT4G27780_ClassII  .......KID AIHGFAREGE VENLLKSIES GIPVNARDSE GRTPLHWAID
 BNAC01G20440D_ClassII  YSVLYLRKID AIHEFAREGE VENLLKSIDS GIPVNAKDSE GRTPLHWAID
 BNAA01G16660D_ClassII  YSVLYLRKID AIHEFAREGE VENLLKSIES GIPVNAKDSE GRTPLHWAID
    AT4G24230_ClassIII  VSGER..... .......... .......... .......... ..........
BNAA01G13710D_ClassIII  VSGEDESSV. .......... .......... .......... ..........
BNAC01G16110D_ClassIII  ..GKDEFPEE .......... .......... .......... ..........
BNAA03G46540D_ClassIII  ..GKTN.... .......... .......... .......... ..........
BNAC07G38820D_ClassIII  ..GKTN.... .......... .......... .......... ..........
     AT3G05420_ClassIV  AETQDKDVVS EDSNTVSVYN QWTAPQTSGQ RPKARYEHGA AVIQDKMYIY
      AIS76194_ClassIV  SETQDKDVVS EVTNSVSVYN QWTAPQTSGQ RPKARYEHGA AVIQDKMYIY
      AIS76199_ClassIV  SETQDKDVIS EVTNSVSVYN QWTAPQTSGQ RPKARYEHGA AVIQDKMYIY
      AIS76196_ClassIV  AETQDKDVVS EDPNTVFVYN QWTAPQTSGQ RPKPRYEHGA AVIQDKMYIY
      AIS76201_ClassIV  AETQDKDVVS EDPNTVSVYN QWTAPQTSGQ RPKARYEHGA AVIQDKMYIY
      AIS76195_ClassIV  AETQDKDVVS EDPNTVSVYN QWTAPQTSGQ RPKARYEHGA AVIQDKMYIY
      AIS76200_ClassIV  AETQDKDVVS EDPNTVSVYN QWTAPQTSGQ RPKARYEHGA AVIQDKMYIY
     AT5G27630_ClassIV  TETQDKDVVL EDPDTVSVYN QWTAPRTSGQ PPKARYQHGA AVIQDKMYMY
      AIS76197_ClassIV  METQDKDVVL ENPNTISVYN QWTAPLTSGH PPKARYEHGA AVIQDKMYMY
      AIS76198_ClassIV  METQDKDVVL ENPNTISVYN QWTAPFTLGQ PPKARYEHGA AVIQDKMYMY
```

```
                         451                                                  500
      AT1G31812_ClassI   .......... .......... .......... .......... ..........
  BNAANNG25690D_ClassI   .......... .......... .......... .......... ..........
  BNAA05G36060D_ClassI   .......... .......... .......... .......... ..........
  BNAA08G07670D_ClassI   .......... .......... .......... .......... ..........
  BNACNNG15340D_ClassI   .......... .......... .......... .......... ..........
     AT5G53470_ClassII   RGHLNVAEAL VDKNADVNAK DNEGQTSLHY AVVCEREALA EFLVKQKADT
 BNAA02G10270D_ClassII   RGHLNVAKAL VDNKADVNAK DNEGQTALHY AVVCEREALA EFLVKQKADT
 BNAC02G44810D_ClassII   RGHLNVAKAL VDKNADVNAK DNEGQTALHY AVVCEREALA EFLVKQKADT
     AT4G27780_ClassII   RGHLNIAKVL VDKNADVNAK DNEGQTPLHY AVVCDREAIA EFLVKQNANT
 BNAC01G20440D_ClassII   RGHFEIAKLL VDKNADVNAK DNEGQTPLHY AVVCDREAIA EFLVKQKANT
 BNAA01G16660D_ClassII   RGHLDIAKLL VDKNADVNAK DNEGQTPLHY AVVCDREAIA EFLVKQKANT
    AT4G24230_ClassIII   .......... .......... .......... .......... ..........
BNAA01G13710D_ClassIII   .......... .......... .......... .......... ..........
BNAC01G16110D_ClassIII   .......... .......... .......... .......... ..........
BNAA03G46540D_ClassIII   .......... .......... .......... .......... ..........
BNAC07G38820D_ClassIII   .......... .......... .......... .......... ..........
      AT3G05420_ClassIV   GGNHNGRYLG DLHVLDLKSW TWSRVETKVA TESQETSTPT LLAPCAGHSL
     AIS76194_ClassIV   GGNHNGRYLG DLHVLDLKNW TWSRVETKVA TES.DETSPT LLSPCAGHSL
     AIS76199_ClassIV   GGNHNGRYLG DLHALDFKNW TWSRVETKVA TES.EETSPT LLSPCAGHTL
     AIS76196_ClassIV   GGNHNGRYLG DLHVLDLKSW TWSRVETKVA TES.EETSPT LLSPCAGHSL
     AIS76201_ClassIV   GGNHNGRYLG DLHVLDLKSW TWSRVETKVA TES.EETSPT LLSPCAGHSL
     AIS76195_ClassIV   GGNHNGRYLG DLHVLDLKSW TWSRVETKVA TES.EETSPT LLSPCAGHSL
     AIS76200_ClassIV   GGNHNGRYLG DLHVLDLKSW TWSRVETKVA TES.EETSPT LLSPCAGHSL
    AT5G27630_ClassIV   GGNHNGRYLG DLHVLDLKNW TWSRVETKVV TGSQETSSPA KLTHCAGHSL
     AIS76197_ClassIV   GGNHNGRYLG DLHVLDLKNW TWSRVETKVV TESQETSSPA TLTHCAGHSL
     AIS76198_ClassIV   GGNHNGRYLG DLHVLDLKNW TWSRVETKVV TESQETSSPA TLTHCAGHSL

                         501                                                  550
      AT1G31812_ClassI   .......... .......... .......... .......... ..........
  BNAANNG25690D_ClassI   .......... .......... .......... .......... ..........
  BNAA05G36060D_ClassI   .......... .......... .......... .......... ..........
  BNAA08G07670D_ClassI   .......... .......... .......... .......... ..........
  BNACNNG15340D_ClassI   .......... .......... .......... .......... ..........
     AT5G53470_ClassII   TIKDEDGNSP LDLCESEWSW MRE.KKDSN. .......... ..........
 BNAA02G10270D_ClassII   SIKDEDGNSP LDLCESEWSW MRE.KKDSD. .......... ..........
 BNAC02G44810D_ClassII   SIKDEDGNSP LDLCESEWSW MRE.KKDSD. .......... ..........
     AT4G27780_ClassII   AAKDEDGNSP LDLCESDWPW IRDSAKQAD. .......... ..........
 BNAC01G20440D_ClassII   ASKDDDGNTP LDLCESDWPW LRETAKQTD. .......... ..........
 BNAA01G16660D_ClassII   ASKDDDGNSP LDLCESDWPW LRETAKQTD. .......... ..........
    AT4G24230_ClassIII   .......... .......... .......... .......... ..........
BNAA01G13710D_ClassIII   .......... .......... .......... .......... ..........
BNAC01G16110D_ClassIII   .......... .......... .......... .......... ..........
BNAA03G46540D_ClassIII   .......... .......... .......... .......... ..........
BNAC07G38820D_ClassIII   .......... .......... .......... .......... ..........
     AT3G05420_ClassIV   IAWDNKLLSI GGHTKDPSES MQVKVFDPHT ITWSMLKTYG KPPVSRGGQS
     AIS76194_ClassIV   IPWDNKFLSI GGHTKDPSES MQVKVFDTHT CTWSMLKTYG KPPVSRGGQS
     AIS76199_ClassIV   IPWDNRLLSV GGHNKDPSES MQVKVFDTHT CTWSMLKTYG KPPVSRGGQS
     AIS76196_ClassIV   IPWDNKLLSI GGHTKNPSES MQVRVFDTHT CTWSMLKTYG KPPVSRGGQS
     AIS76201_ClassIV   IPWDNKLLSI GGHTKDHSES MQVKVFDTHT STWSMLKTYG KPPVSRGGQS
     AIS76195_ClassIV   IPWDNKLLSI GGHTKDHSES MQVKVFDTHT STWSMLKTYG KPPVSRGGQS
     AIS76200_ClassIV   IPWDNKLLSI GGHTKDHSES MQVKVFDTHT STWSMLKTYG KPPVSRGGQS
    AT5G27630_ClassIV   IPWDNQLLSI GGHTKDPSES MPVMVFDLHC CSWSILKTYG KPPISRGGQS
     AIS76197_ClassIV   IPWDNKLLSI GGHAKDPSES ILVKVFDLHT CTWSILKTDG KPPISRGGQS
     AIS76198_ClassIV   IPWDNKLLSI GGHAKDPSES ILVKVFDLHT CIWSILKTDG KPPISRGGQS

                         551                                                  600
```

```
       AT1G31812_ClassI  .......... .......... .......... .......... ..........
   BNAANNG25690D_ClassI  .......... .......... .......... .......... ..........
   BNAA05G36060D_ClassI  .......... .......... .......... .......... ..........
   BNAA08G07670D_ClassI  .......... .......... .......... .......... ..........
   BNACNNG15340D_ClassI  .......... .......... .......... .......... ..........
      AT5G53470_ClassII  .......... .......... .......... .......... ..........
  BNAA02G10270D_ClassII  .......... .......... .......... .......... ..........
  BNAC02G44810D_ClassII  .......... .......... .......... .......... ..........
      AT4G27780_ClassII  .......... .......... .......... .......... ..........
  BNAC01G20440D_ClassII  .......... .......... .......... .......... ..........
  BNAA01G16660D_ClassII  .......... .......... .......... .......... ..........
     AT4G24230_ClassIII  .......... .......... .......... .......... ..........
 BNAA01G13710D_ClassIII  .......... .......... .......... .......... ..........
 BNAC01G16110D_ClassIII  .......... .......... .......... .......... ..........
 BNAA03G46540D_ClassIII  .......... .......... .......... .......... ..........
 BNAC07G38820D_ClassIII  .......... .......... .......... .......... ..........
      AT3G05420_ClassIV  VTMVGKTLVI FGGQDAKRSL LNDLHILDLD TMTWDEIDAV GVSPSPRSDH
       AIS76194_ClassIV  VTVVGKTLVI FGGQDAKRSL LNDLHVLDLE TMTWDEIDSI GASPSPRSDH
       AIS76199_ClassIV  VTVVGKTLVI FGGQDAKRSL LNDLHVLDLE TMTWDEIDSI GASPSPRSDH
       AIS76196_ClassIV  VTVVGKTLVI FGGQDAKRSL LNDLHVLDLE TMTWDEVDSI GVSPSPRSDH
       AIS76201_ClassIV  VTVVGKTLVI FGGQDAKRSL LNDLHVLDLE TMTWDEIDSI GVSPSPRSDH
       AIS76195_ClassIV  VTVVGKTLVI FGGQDAKRSL LNDLHVLDLE TMTWDEIDAL GVSPSPRSDH
       AIS76200_ClassIV  VTVVGKTLVI FGGQDAKRSL LNDLHVLDLE TMTWDEIDAL GVSPSPRSDH
      AT5G27630_ClassIV  VTLVGKSLVI FGGQDAKRSL LNDLHILDLD TMTWEEIDAV GSPPTPRSDH
       AIS76197_ClassIV  VTLVGKKLVI FGGQNVNKSL LNDLHLLDLD TMTWDEIDAV GSPPSPRSDH
       AIS76198_ClassIV  VTLVGKKLVI FGGQDVNKSL LNDLHLLDLD TMTWDEIDAV GSPPSPRSDH

                         601                                                650
       AT1G31812_ClassI  .......... .......... .......... .......... ..........
   BNAANNG25690D_ClassI  .......... .......... .......... .......... ..........
   BNAA05G36060D_ClassI  .......... .......... .......... .......... ..........
   BNAA08G07670D_ClassI  .......... .......... .......... .......... ..........
   BNACNNG15340D_ClassI  .......... .......... .......... .......... ..........
      AT5G53470_ClassII  .......... .......... .......... .......... ..........
  BNAA02G10270D_ClassII  .......... .......... .......... .......... ..........
  BNAC02G44810D_ClassII  .......... .......... .......... .......... ..........
      AT4G27780_ClassII  .......... .......... .......... .......... ..........
  BNAC01G20440D_ClassII  .......... .......... .......... .......... ..........
  BNAA01G16660D_ClassII  .......... .......... .......... .......... ..........
     AT4G24230_ClassIII  .......... .......... .......... .......... ..........
 BNAA01G13710D_ClassIII  .......... .......... .......... .......... ..........
 BNAC01G16110D_ClassIII  .......... .......... .......... .......... ..........
 BNAA03G46540D_ClassIII  .......... .......... .......... .......... ..........
 BNAC07G38820D_ClassIII  .......... .......... .......... .......... ..........
      AT3G05420_ClassIV  AAAVHAERFL LIFGGGSHAT CFDDLHVLDL QTMEWSRPAQ QGDAPTPRAG
       AIS76194_ClassIV  AAAVHAERYL LIFGGGSHAT CFGDLHVLDL QTMEWSRPAQ QGEAPTPRSG
       AIS76199_ClassIV  AAAVHAERYL LIFGGGSHAT CFGDLHVLDL QTMEWSRPAQ QGEAPTPRSG
       AIS76196_ClassIV  AAAVHAERYL LIFGGGSHAT CFGDLHVLDL QTMEWSRPAQ QGEAPTPRSG
       AIS76201_ClassIV  AAAVHAERYL LIFGGGSHAT CFGDLHVLDL QTMEWSRPAQ QGEAPTPRSG
       AIS76195_ClassIV  AAAVHAERYL LIFGGGSHAT CFDDLHVLDL QTMEWSRPAQ QGEVPTPRSG
       AIS76200_ClassIV  AAAVHAERYL LIFGGGSHAT CFDDLHVLDL QTMEWSRPAQ QGEVPTPRSG
      AT5G27630_ClassIV  AAAVHAERYL LIFGGGSHAT CFDDLHVLDL QTMEWSRHTQ QGDAPTPRAG
       AIS76197_ClassIV  AAAVHAERYL LIFGGGSHTN CFSDLHVLDL QTMEWSRHAQ QGEAPTPRAG
       AIS76198_ClassIV  AAAVHAERYL LIFGGGSHTN CFSDLHVLDL QTMEWSRHAQ QGDVPSPRAG

                         651                                                700
       AT1G31812_ClassI  .......... .......... .......... .......... ..........
```

```
BNAANNG25690D_ClassI  .......... .......... .......... .......... ..........
BNAA05G36060D_ClassI  .......... .......... .......... .......... ..........
BNAA08G07670D_ClassI  .......... .......... .......... .......... ..........
BNACNNG15340D_ClassI  .......... .......... .......... .......... ..........
AT5G53470_ClassII  .......... .......... .......... .......... ..........
BNAA02G10270D_ClassII  .......... .......... .......... .......... ..........
BNAC02G44810D_ClassII  .......... .......... .......... .......... ..........
AT4G27780_ClassII  .......... .......... .......... .......... ..........
BNAC01G20440D_ClassII  .......... .......... .......... .......... ..........
BNAA01G16660D_ClassII  .......... .......... .......... .......... ..........
AT4G24230_ClassIII  .......... .......... .......... .......... ..........
BNAA01G13710D_ClassIII  .......... .......... .......... .......... ..........
BNAC01G16110D_ClassIII  .......... .......... .......... .......... ..........
BNAA03G46540D_ClassIII  .......... .......... .......... .......... ..........
BNAC07G38820D_ClassIII  .......... .......... .......... .......... ..........
AT3G05420_ClassIV  HAGVTIGENW FIVGGGDNKS GASESVVLNM STLAWSVVAS VQGRVPLASE
AIS76194_ClassIV  HAGVTNGENW FIVGGGDNKS GASESVVLNM STLTWSVVAS VQGRVPLASE
AIS76199_ClassIV  HAGVTNGENW FIVGGGDNKS GASESVVLNM STLTWSVVAS VEGRVPLASE
AIS76196_ClassIV  HAGVTIGENW FIVGGGDNKS GASESVVLNM STLSWSVIAS VEGCVPLASE
AIS76201_ClassIV  HAGVTIGENW FIVGGGDNKS GASESVVLNM STLSWSVIAS VEGCVPLASE
AIS76195_ClassIV  HAGVTIGENW FIVGGGDNKS GASESVVLNM STLTWSVVAS VQGRVPLASE
AIS76200_ClassIV  HAGVTIGENW FIVGGGDNKS GASESVVLNM STLTWSVVAS VQGRVPLASE
AT5G27630_ClassIV  HAGVTIGENW YIVGGGDNKS GASKTVVLNM STLAWSVVTS VQEHVPLASE
AIS76197_ClassIV  HAGVTIGENW FIVGGGDNKS GACETVVLNM STLAWSVLTT VQGGAPLASE
AIS76198_ClassIV  HAGVTIGENW FIVGGGDNKS GACETVVLNM STLAWSVLTS VQGGVPLASE

                  701                                                 750
AT1G31812_ClassI  .......... .......... .......... .......... ..........
BNAANNG25690D_ClassI  .......... .......... .......... .......... ..........
BNAA05G36060D_ClassI  .......... .......... .......... .......... ..........
BNAA08G07670D_ClassI  .......... .......... .......... .......... ..........
BNACNNG15340D_ClassI  .......... .......... .......... .......... ..........
AT5G53470_ClassII  .......... .......... .......... .......... ..........
BNAA02G10270D_ClassII  .......... .......... .......... .......... ..........
BNAC02G44810D_ClassII  .......... .......... .......... .......... ..........
AT4G27780_ClassII  .......... .......... .......... .......... ..........
BNAC01G20440D_ClassII  .......... .......... .......... .......... ..........
BNAA01G16660D_ClassII  .......... .......... .......... .......... ..........
AT4G24230_ClassIII  .......... .......... .......... .......... ..........
BNAA01G13710D_ClassIII  .......... .......... .......... .......... ..........
BNAC01G16110D_ClassIII  .......... .......... .......... .......... ..........
BNAA03G46540D_ClassIII  .......... .......... .......... .......... ..........
BNAC07G38820D_ClassIII  .......... .......... .......... .......... ..........
AT3G05420_ClassIV  GLSLVVSSYN GEDVLVAFGG YNGRYNNEIN LLKPSHKSTL .QTKTLEAPL
AIS76194_ClassIV  GLSLVVSSYN GEDVLVAFGG YNGRYNNEIN LLKPSHKSTL .QPKTLEAPL
AIS76199_ClassIV  GLSLVVSSYN GEDVLVAFGG YNGRYNNEIN LLKPSHKSTL .QPKTLEPPL
AIS76196_ClassIV  GLSLVVSSYN GEDVLVAFGG YNGRYNNEIN LLKPSHKSTL .QQKTVEAPL
AIS76201_ClassIV  GLSLVVSSYN GEDVLVAFGG YNGRYNNEIN LLKPSHKSTL .QQKTVEAPL
AIS76195_ClassIV  GLSLVVSSYN GEDVLVAFGG YNGRYNNEVN LLKPSHKSTL .QPKTLEDPL
AIS76200_ClassIV  GLSLVVSSYN GEDVLVAFGG YNGRYNNEVN LLKPSHKSTL .QPKTLEDPL
AT5G27630_ClassIV  GLSLVVSSYN GEDIVVAFGG YNGHYNNEVN VLKPSHKSSL KSKIMGASAV
AIS76197_ClassIV  GLSLVVSSYN GEDVIVAFGG YNGRYNNEVN VLKPSHKSSL KSKIMEASPV
AIS76198_ClassIV  GLSLVVSAYN GEDVIVAFGG YNGRYNNEVN VLKPSHKSSL KSKIMEASPV

                  751                                                 800
AT1G31812_ClassI  .......... .......... .......... .......... ..........
BNAANNG25690D_ClassI  .......... .......... .......... .......... ..........
```

```
  BNAA05G36060D_ClassI  .......... .......... .......... .......... ..........
  BNAA08G07670D_ClassI  .......... .......... .......... .......... ..........
  BNACNNG15340D_ClassI  .......... .......... .......... .......... ..........
     AT5G53470_ClassII  .......... .......... .......... .......... ..........
 BNAA02G10270D_ClassII  .......... .......... .......... .......... ..........
 BNAC02G44810D_ClassII  .......... .......... .......... .......... ..........
     AT4G27780_ClassII  .......... .......... .......... .......... ..........
 BNAC01G20440D_ClassII  .......... .......... .......... .......... ..........
 BNAA01G16660D_ClassII  .......... .......... .......... .......... ..........
    AT4G24230_ClassIII  .......... .......... .......... .......... ..........
BNAA01G13710D_ClassIII  .......... .......... .......... .......... ..........
BNAC01G16110D_ClassIII  .......... .......... .......... .......... ..........
BNAA03G46540D_ClassIII  .......... .......... .......... .......... ..........
BNAC07G38820D_ClassIII  .......... .......... .......... .......... ..........
     AT3G05420_ClassIV  PGSLSAVNNA TTRDIESEVE VSQEGRVREI VMDNVNPGSK VEGNSERIIA
      AIS76194_ClassIV  PVSLSAVNNA TTRDIESEVE VSQESRVREI LMDNANAGSK VEGNSEHIIA
      AIS76199_ClassIV  PVSLSAVNNA TTRDIESEVE VSQESRVREI LMDNANAGSE VEGNSEHIIA
      AIS76196_ClassIV  PGSLSAVNNA TTRDIESE.. VSQEGRVREI AMDNVSPGSK VEVNNEHIIA
      AIS76201_ClassIV  PGSLSAVNNA TTRDIESE.. VSQEGRVREI AMDNVSPGSK VEVNNEHIIA
      AIS76195_ClassIV  PVSLSAVNNA TTRDIESEVE VSQESRVREI VMDNVNPGSK VEGNNERIIE
      AIS76200_ClassIV  PVSLSAVNNA TTRDIESEVE VSQESRVREI VMDNVNPGSK VEGNNERIIE
     AT5G27630_ClassIV  PDSFSAVNNA TTRDIESEI. .......... .........K VEGKADRIIT
      AIS76197_ClassIV  RDSVSAVNNA TTRDIEFEIG VSQESKVREI VMDNVNSGSK VEGKSERIIT
      AIS76198_ClassIV  RDSVSAVNNA TTRDIESEIG VSQESKVREI VVDNVNSGSK VEGKSERIIT

                        801                                                850
      AT1G31812_ClassI  .......... .......... .......... .......... ..........
  BNAANNG25690D_ClassI  .......... .......... .......... .......... ..........
  BNAA05G36060D_ClassI  .......... .......... .......... .......... ..........
  BNAA08G07670D_ClassI  .......... .......... .......... .......... ..........
  BNACNNG15340D_ClassI  .......... .......... .......... .......... ..........
     AT5G53470_ClassII  .......... .......... .......... .......... ..........
 BNAA02G10270D_ClassII  .......... .......... .......... .......... ..........
 BNAC02G44810D_ClassII  .......... .......... .......... .......... ..........
     AT4G27780_ClassII  .......... .......... .......... .......... ..........
 BNAC01G20440D_ClassII  .......... .......... .......... .......... ..........
 BNAA01G16660D_ClassII  .......... .......... .......... .......... ..........
    AT4G24230_ClassIII  .......... .......... .......... .......... ..........
BNAA01G13710D_ClassIII  .......... .......... .......... .......... ..........
BNAC01G16110D_ClassIII  .......... .......... .......... .......... ..........
BNAA03G46540D_ClassIII  .......... .......... .......... .......... ..........
BNAC07G38820D_ClassIII  .......... .......... .......... .......... ..........
     AT3G05420_ClassIV  TIKSEKEELE ASLNKERMQT LQLRQELGEA ELRNTDLYKE LQSVRGQLAA
      AIS76194_ClassIV  IIKSEKEELE ASPNKEQMQT LQLRQELAEG ESRNTDLNKE LQSVRGQLAT
      AIS76199_ClassIV  ILKSEKEELE ASLNKEQMQT LQLRQELAEG ESRNTDLNKE LQSVRGQLAT
      AIS76196_ClassIV  TLKSEKEELE ASLNKEKLQT QQLRQELSEA ESRNTDLHKE LQSDRGQLQA
      AIS76201_ClassIV  TLKSEKEELE ASLNKEKLQT QQLRQELSEA ESRNTDLHKE LQSDRGQLQA
      AIS76195_ClassIV  TLKAEKEELE LSLNKEKTQT LQLRQELADG ESRNADLYKE LHSVRSQLAT
      AIS76200_ClassIV  TLKAEKEELE LSLNKEKTQT LQLRQELADG ESRNADLYKE LHSVRSQLAT
     AT5G27630_ClassIV  TLKSEKEEVE ASLNKEKIQT LQLKEELAEI DTRNTELYKE LQSVRNQLAA
      AIS76197_ClassIV  SLRSEKEELE ASLSKEKIQT LQLKEELTET ETRSTELYKE LHSVRSQLAA
      AIS76198_ClassIV  SLRSEKEELE ASLSKEKIQT LQLKEELTET ETRSAELYKE LHSVRSQLAA

                        851                                                900
      AT1G31812_ClassI  .......... .......... .......... .......... ..........
  BNAANNG25690D_ClassI  .......... .......... .......... .......... ..........
  BNAA05G36060D_ClassI  .......... .......... .......... .......... ..........
```

```
   BNAA08G07670D_ClassI  .......... .......... .......... .......... ..........
   BNACNNG15340D_ClassI  .......... .......... .......... .......... ..........
      AT5G53470_ClassII  .......... .......... .......... .......... ..........
  BNAA02G10270D_ClassII  .......... .......... .......... .......... ..........
  BNAC02G44810D_ClassII  .......... .......... .......... .......... ..........
      AT4G27780_ClassII  .......... .......... .......... .......... ..........
  BNAC01G20440D_ClassII  .......... .......... .......... .......... ..........
  BNAA01G16660D_ClassII  .......... .......... .......... .......... ..........
     AT4G24230_ClassIII  .......... .......... .......... .......... ..........
 BNAA01G13710D_ClassIII  .......... .......... .......... .......... ..........
 BNAC01G16110D_ClassIII  .......... .......... .......... .......... ..........
 BNAA03G46540D_ClassIII  .......... .......... .......... .......... ..........
 BNAC07G38820D_ClassIII  .......... .......... .......... .......... ..........
      AT3G05420_ClassIV  EQSRCFKLEV DVAELRQKLQ TLETLQKELE LLQRQKA.AS EQAA..MNAK
       AIS76194_ClassIV  EQSRCFKLEV DVAELRQKLQ TLETLQKELE LLQRQKA.VS EQAAT..NTK
       AIS76199_ClassIV  EQSRCFKLEV DVAELRQKLQ TLETLQKELE LLQRQKA.VS EQAAT..NTK
       AIS76196_ClassIV  EQSRCFKLEV DVAELRQKLQ TLETLQKELE LLQRQKA.AS EQAST..NAK
       AIS76201_ClassIV  EQSRCFKLEV DVAELRQKLQ TLETLQKELE LLQRQKA.AS EQAST..NAK
       AIS76195_ClassIV  EQSRCFKLEV DVAELRQKLQ TLETLQKELE LLQRQKA.AS EQAA..MNAK
       AIS76200_ClassIV  EQSRCFKLEV DVAELRQKLQ TLETLQKELE LLQRQKA.AS EQAA..MNAK
      AT5G27630_ClassIV  EQSRCFKLEV EVAELRQKLQ TMETLQKELE LLQRQRAVAS EQAAT.MNAK
       AIS76197_ClassIV  EQSRCFKLEV EVAEVRQKLQ TMETLEKELE LLHRQRAVAS EQAAVNMNGK
       AIS76198_ClassIV  EQSRCFKLEV EVAEVRQKLQ TMETLEKELE LLHRQRAVAS EQAAVNMNGK

                         901                    923
       AT1G31812_ClassI  .......... .......... ...
   BNAANNG25690D_ClassI  .......... .......... ...
   BNAA05G36060D_ClassI  .......... .......... ...
   BNAA08G07670D_ClassI  .......... .......... ...
   BNACNNG15340D_ClassI  .......... .......... ...
      AT5G53470_ClassII  .......... .......... ...
  BNAA02G10270D_ClassII  .......... .......... ...
  BNAC02G44810D_ClassII  .......... .......... ...
      AT4G27780_ClassII  .......... .......... ...
  BNAC01G20440D_ClassII  .......... .......... ...
  BNAA01G16660D_ClassII  .......... .......... ...
     AT4G24230_ClassIII  .......... .......... ...
 BNAA01G13710D_ClassIII  .......... .......... ...
 BNAC01G16110D_ClassIII  .......... .......... ...
 BNAA03G46540D_ClassIII  .......... .......... ...
 BNAC07G38820D_ClassIII  .......... .......... ...
      AT3G05420_ClassIV  RQGSGGVWGW LAGSPQEKDD DSP
       AIS76194_ClassIV  RQSSGGVWGW LAGSPQEK.D DSP
       AIS76199_ClassIV  RQSSGGVWGW LAGSPHEK.D DSP
       AIS76196_ClassIV  RQSSGGVWGW LAGSPQEKDD DSP
       AIS76201_ClassIV  RQSSGGVWGW LAGSPQEKDD DSP
       AIS76195_ClassIV  RQSSGGVWGW LAGSPQEK.D DSP
       AIS76200_ClassIV  RQSSGGVWGW LAGSPQEK.D DSP
      AT5G27630_ClassIV  RQSSGGVWGW LAGTPPPKT. ...
       AIS76197_ClassIV  PQSSGGVWGW LAGTPPPKT. ...
       AIS76198_ClassIV  RQSSGGVWGW LAGTPPPKT. ...
```
