# Supplementary material for: Genome-wide identification and Phylogenic analysis of kelch motif containing ACBP in Brassica napus
Source: BMC Genomics. 2015 Jul 9;16(1):512. doi: 10.1186/s12864-015-1735-6 (PMC4497377; doi:10.1186/s12864-015-1735-6)
Supplement: Additional file 7: Table S1. — Primer sequences used to clone kelch motif BnACBPs. [file 12864_2015_1735_MOESM7_ESM.doc]

**Supplementary Table 1 Primer sequences used to clone kelch motif *Bn*ACBPs.**

| **Homologue genes** | **Primers** |
| --- | --- |
| Bra040219 | 5' GGAATTCATGGCTATGGCTAGAGCAACATCTG 3'  5' TCCCCCGGGCTATGGCGAATCATCATCCTTCTCC 3' |
| Bra039439 | 5' GGAATTCATGGCTATGGCTAGAGCAACATCTG 3'  5' TCCCCCGGGTCATGGCGAATCATCTTTCTCCTGAG 3' |
| Bra020582 | 5' GGAATTCATGGCGAAGGCGAGCGCTAC 3'  5' TCCCCCGGGTCAAGTTTTTGGCGGAGGAGTTCCAG 3' |
| Bra001147 | 5' GGAATTCATGGCTATAACAAGGGCCACATCTG 3'  5' TCCCCCGGGTCATGGAGAATCATCTTTCTCCTGAGGG 3' |
| Bol034106 | 5' GGAATTCATGGCTATAACTAGGGCCACATCTG 3'  5' TCCCCCGGGTCATGGCGAATCATCTTTCTCATGAGG 3' |
| Bol012774 | 5' GGAATTCATGGCGAAGCAGAGCGCTAC 3'  5' TCCCCCGGGTCAAGTTTTTGGCGGAGGAGTTCCA 3' |
| Bol002733 | 5' GGAATTCATGGCTATGGCTAGAGCAACATCTG 3'  5' TCCCCCGGGTTATGGCGAATCATCATCCTTCTCC 3' |
| Bol001638 | 5' GGAATTCATGGCTATGGCTAGAGCAACATCTG 3'  5' TCCCCCGGGTCATGGCGAATCATCTTTCTCCTGA 3' |
