# Supplementary material for: Genome-wide identification and Phylogenic analysis of kelch motif containing ACBP in Brassica napus
Source: BMC Genomics. 2015 Jul 9;16(1):512. doi: 10.1186/s12864-015-1735-6 (PMC4497377; doi:10.1186/s12864-015-1735-6)
Supplement: Additional file 8: Table S2. — List of plants species used in phylogenetic analysis. [file 12864_2015_1735_MOESM8_ESM.doc]

**Supplementary Table 2 List of plants species used in phylogenetic analysis.**

| **Species** | **Class I** | **Class II** | **Class III** | **Class IV** |
| --- | --- | --- | --- | --- |
| *Arabidopsis*  *thaliana* | At1g31812 | At5g53470  At4g27780 | At4g24230 | At3g05420  At5g27630 |
| *Arabidopsis*  *lyrata* | XP_002893688 | - | XP_002867674 | XP_002882412  XP_002874360 |
| *Brassica*  *rapa* | Bra023206  Bra033875  Bra038439 | Bra022656  Bra026307 | Bra013778  Bra019240 | Bra001147  Bra020582  Bra040219  Bra039439 |
| *Brassica*  *oleracea* | Bol005980  Bol027060  Bol038626 | Bol017188  Bol013113 | Bol009564  Bol042158 | Bol001638  Bol002733  Bol012774  Bol034106 |
| *Brassica*  *napus* | BnaAnng25690D  BnaA05g36060D  BnaA08g07670D  BnaCnng15340D | BnaA02g10270D  BnaA01g16660D  BnaC02g44810D  BnaC01g20440D | BnaA01g13710D  BnaA03g46540D  BnaC01g16110D  BnaC07g38820D | AIS76194  AIS76195  AIS76196  AIS76197  AIS76198  AIS76199  AIS76200  AIS76201 |
| *Oryza*  *sativa* | Os08g06550  Os03g37960  Os06g02490 | Os04g58550 | Os03g14000 | Os03g61930 |
| *Zea*  *mays* | Zm10g09880  Zm09g06330 | [Zm10g26640](http://www.maizesequence.org/Zea_mays/Transcript/Summary?t=GRMZM2G173636_T01) | [Zm01g09480](http://www.maizesequence.org/Zea_mays/Transcript/Summary?t=GRMZM2G108138_T01) | [Zm01g58820](http://www.maizesequence.org/Zea_mays/Transcript/Summary?t=GRMZM2G053803_T01) |

**Algae outgroup:**

*Chlorella sp.* ACBP: Aiu80189
